# Genome-wide compendium and functional assessment of *in vivo* heart enhancers

**DOI:** 10.1038/ncomms12923

**Published:** 2016-10-05

**Authors:** Diane E. Dickel, Iros Barozzi, Yiwen Zhu, Yoko Fukuda-Yuzawa, Marco Osterwalder, Brandon J. Mannion, Dalit May, Cailyn H. Spurrell, Ingrid Plajzer-Frick, Catherine S. Pickle, Elizabeth Lee, Tyler H. Garvin, Momoe Kato, Jennifer A. Akiyama, Veena Afzal, Ah Young Lee, David U. Gorkin, Bing Ren, Edward M. Rubin, Axel Visel, Len A. Pennacchio

**Affiliations:** 1 Functional Genomics Department, Lawrence Berkeley National Laboratory, 1 Cyclotron Road, Berkeley, California 94720, USA; 2 Ludwig Institute for Cancer Research, 9500 Gilman Drive, La Jolla, California 92093, USA; 3 Department of Cellular and Molecular Medicine, University of California, San Diego School of Medicine, La Jolla, California 92093, USA; 4 U.S. Department of Energy Joint Genome Institute, Walnut Creek, California 94598, USA; 5 School of Natural Sciences, University of California, Merced, Merced, California 95343, USA

## Abstract

Whole-genome sequencing is identifying growing numbers of non-coding variants in human disease studies, but the lack of accurate functional annotations prevents their interpretation. We describe the genome-wide landscape of distant-acting enhancers active in the developing and adult human heart, an organ whose impairment is a predominant cause of mortality and morbidity. Using integrative analysis of >35 epigenomic data sets from mouse and human pre- and postnatal hearts we created a comprehensive reference of >80,000 putative human heart enhancers. To illustrate the importance of enhancers in the regulation of genes involved in heart disease, we deleted the mouse orthologs of two human enhancers near cardiac myosin genes. In both cases, we observe *in vivo* expression changes and cardiac phenotypes consistent with human heart disease. Our study provides a comprehensive catalogue of human heart enhancers for use in clinical whole-genome sequencing studies and highlights the importance of enhancers for cardiac function.

## Abstract (web summary)

Identification of non-coding variants has outstripped our ability to annotate and interpret them. Dickel *et al*. present a compendium of over 80,000 putative human heart enhancers and demonstrate that two conserved enhancers are required for proper cardiac function in mice.

## Graphical abstract

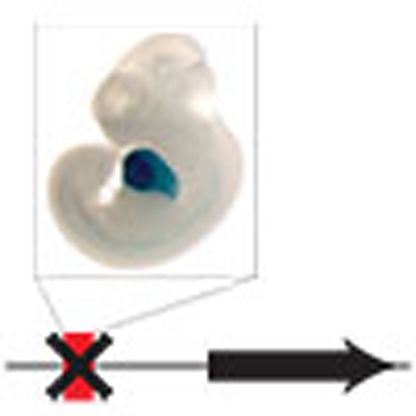

Cardiovascular disease is the most common cause of death worldwide^1^. Diseases of the heart include a spectrum of adult-onset conditions, as well as congenital phenotypes that collectively represent the most common category of severe birth defects^2^. Causes of heart disease include environmental risk factors^1^, common variants with moderate effect sizes^3^, and rare and *de novo* mutations that cause familial cases with Mendelian inheritance patterns^3^. In particular for the latter category, candidate gene sequencing has proven powerful for obtaining molecular diagnoses. For example, for familial hypertrophic cardiomyopathy, candidate gene sequencing identifies a clear genetic cause in ∼60% of patients^4^. Nevertheless, this approach is by design limited to the coding sequence of candidate genes and fails to identify non-coding mutations. Whole-genome sequencing (WGS) can in principle detect non-coding mutations and is becoming increasingly adopted for patients with unexplained heart disease^5^. However, early WGS studies illustrate major challenges in the interpretation of non-coding variants, and particularly of rare non-coding variants^6^. In the absence of accurate annotations linking non-coding loci to *in vivo* functions, non-coding WGS findings are largely uninterpretable and, thereby, most cases with non-coding mutations remain unresolved.

To address the pressing need for a high-quality, genome-wide annotation of functional non-coding sequences active in the developing and adult heart, in the present study we describe a comprehensive catalogue of more than 80,000 candidate distant-acting cardiac enhancers (Fig. 1). Enhancers are a major category of non-coding regulatory elements that activate gene expression from a distance in a cell type-specific^7^ and temporally restricted^8^ manner. They are hypothesized to play a major role in development and disease, and sequence variants that alter enhancer function are associated with a variety of human phenotypes (for example, refs 9, 10, 11). We derived the heart enhancer compendium from more than three dozen epigenomic data sets mapping enhancer-associated chromatin marks in developing and adult heart tissue from mice and humans. This catalogue of human heart enhancers can be easily and immediately implemented in human disease studies, and to further facilitate its utilization in clinical studies, we provide confidence scores for each predicted enhancer that correlate strongly with *in vivo* validation rates. We find that more than 2,000 human variants implicated in heart-related phenotypes through genome-wide association studies (GWAS), either directly as lead variants or indirectly by linkage disequilibrium (LD), fall into putative heart enhancers. Anticipating downstream validation of WGS studies, which will likely focus first on regulatory sequences near genes already implicated in disease, we experimentally validated putative enhancers and provide *in vivo* characterization of more than 20 novel cardiovascular enhancers near known heart disease genes. Finally, as there remains a limited understanding of the general phenotypic impact of lost or impaired enhancer function, we deleted two enhancers near heart disease genes in mice. In both cases, we observed loss of target gene expression, as well as cardiac phenotypes consistent with heart disease in humans. Our results highlight the functional importance of enhancers for normal heart function, as well as the potential contribution of enhancer mutations to heart disease.

## Results

### Genome-wide mapping of heart enhancers

Genome-wide profiling of enhancer-associated proteins and histone modifications such as p300/CBP or H3K27ac via chromatin immunoprecipitation (ChIP)-seq directly applied to primary tissue is a powerful approach for the identification of *in vivo* enhancers^12^,^13^. Initial application of this technique to cardiac tissue samples established the general utility of the method for the identification of heart enhancers, but detected only modestly sized sets of candidate enhancers due to limited sampling^14^,^15^. To generate a comprehensive genome-wide catalogue of cardiac enhancers in the human genome that can easily be incorporated into human disease studies, we integrated epigenomic data from multiple developmental stages and all major anatomical subregions of the heart. In total, we examined >35 genome-wide p300/CBP and/or H3K27ac profiles from different *ex vivo* cardiac tissue samples, nearly all of which are known to be or presumed to be normal (see Table 1 and Supplementary Table 1 for tissue information and data sources)^8^,^14^,^15^,^16^,^17^,^18^,^19^. The sampled conditions include prenatal human heart, major anatomical subregions of childhood and adult human hearts, and a closely spaced developmental time series of prenatal and postnatal mouse heart (Table 1). Although the inclusion of mouse samples could create bias towards the prediction of more highly conserved enhancers (see Supplementary Note 1), it allows for better discovery of enhancers active exclusively during prenatal development, a timespan for which there is only a single human data set considered for analysis. To enable integrative analysis across samples and antibodies, all raw data was analysed using a uniform processing pipeline (see the ‘Methods' section, Supplementary Fig. 1). Because H3K27ac and p300 are associated with both enhancer and promoter sequences^20^,^21^, we excluded peaks overlapping promoters, defined as those centred within 1.5 kb of a gene transcription start site (TSS; promoters and their scores are included separately in Supplementary Data 1 but were not further evaluated). Peaks identified in mouse samples were mapped to the human genome (see Supplementary Table 2). Merging the peaks from all data sets resulted in a single list of 82,119 unique candidate heart enhancers in the human genome (Supplementary Data 2). Nearly all (>95%) of the putative enhancers were <10 kb in size (Supplementary Fig. 2a), and most (85%) were smaller than 5 kb. A total of 3,677 candidate enhancer regions >10 kb were identified, consistent with previous observations of subsets of very long enhancers that may form central nodes of tissue-specific regulatory networks (Supplementary Note 2)^22^.

**Figure 2:**
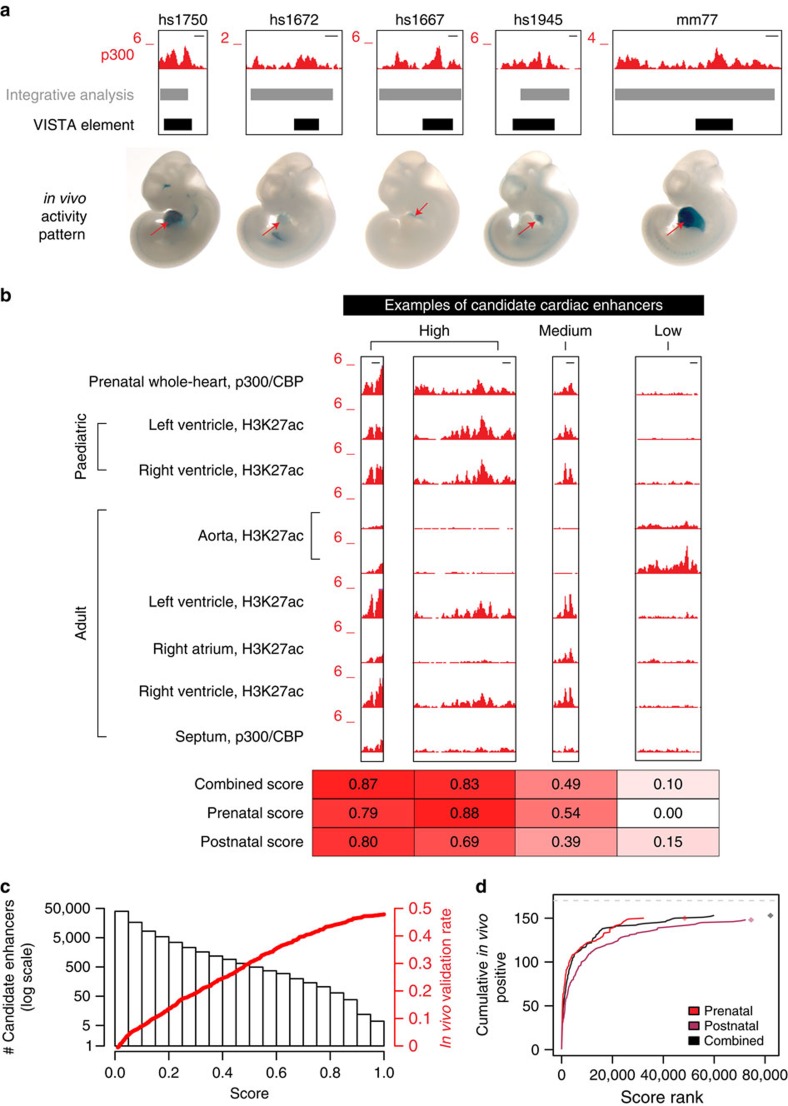
Integrative analysis of heart ChIP-seq data identifies >80,000 heart enhancers. (**a**) Examples of heart enhancers identified through this analysis that had previously been validated in transgenic mouse assays^24^. For each locus, we show the raw p300/CBP ChIP-seq signal from foetal human heart, the locations of the integrative analysis-identified enhancer and the tested element, and a representative E11.5 embryo. The heart is indicated by a red arrow. hs/mm numbers indicate the VISTA identifier. Scale bars, 1 kb. (**b**) Representative examples of putative enhancers with high, medium and low levels of support. Raw ChIP-seq data from all human heart samples analysed is shown, along with the corresponding confidence scores for each locus. (**c**) Histogram of combined scores for all 82,119 putative heart enhancers identified by the integrative analysis (left axis). Superimposed (red) is the retrospective heart enhancer validation rate versus combined score for all *in vivo* tested sites that overlap scored regions (see the ‘Methods' section). (**d**) Cumulative proportion of *in vivo*-validated heart enhancers captured by score rank. Dashed gray line indicates the total number of validated heart enhancers considered (170). Rhombi indicate the total number of scored loci in each category.

Two initial lines of evidence support that this catalogue is comprised of cardiac-specific enhancers. First, gene ontology analysis^23^ showed that the identified regions are highly enriched near genes with relevant functions (Supplementary Table 3). For example, 11 out of the 12 most enriched human phenotypes represent cardiovascular conditions (Table 2). In addition, 152 out of 170 (89%) heart enhancers reported in the VISTA Enhancer Browser, a large collection of *in vivo* enhancers validated in transgenic mice^24^, are identified as candidate heart enhancers in the present catalogue (Fig. 2a, Supplementary Fig. 2b, Supplementary Data 2). This high rediscovery rate highlights the sensitivity of the approach and suggests that most known heart enhancers are recovered by the integrative analysis in the present study.

Although p300 and H3K27ac are strong predictors of enhancer activity, there have been recent reports of improved enhancer prediction using various additional criteria (for example, transcription factor (TF) binding and DNase hypersensitivity (DHS))^25^ and/or supervised analysis methods trained on experimentally validated enhancer sets^26^,^27^. To assess whether additional types of data sets or methods would substantially improve enhancer prediction, we first performed a similar integrative analysis using available human and mouse heart DHS and TF data sets (data sets and their references are listed in Supplementary Table 4). Overall, DHS and TF ChIP-seq identify sets of loci that substantially overlap those captured by H3K27ac and p300 (Supplementary Fig. 3a,b). Those sites displaying DHS or TF binding in the absence of H3K27ac or p300 are not associated with cardiac-specific function, suggesting they do not identify cardiac enhancers missed by our integrative H3K27ac and p300 analysis (Supplementary Fig. 3c,d). We additionally found that our unsupervised approach performs with an accuracy similar to EMERGE (ref. 26) and EnhancerFinder (ref. 27), supervised methods for enhancer prediction (Supplementary Table 5).

Our analysis, using a large number of data sets and relatively permissive criteria for the identification of candidate heart enhancers, identifies a considerable proportion of the human genome (264 Mb or ∼8% of the total genome) as potential enhancers under at least one of the conditions studied. However, individual predicted enhancer sequences vary substantially in the strength of the supporting evidence, which includes the intensity of ChIP-seq signal in individual data sets (peak scores), as well as recurrent observation of the same peak across multiple cardiac source tissues (Fig. 2b, top). We hypothesized that these differences provide a means to distinguish higher- from lower-confidence predictions, allowing for rational experimental prioritization of candidate sequences. Such evidence-based ranking is critical because WGS studies identify far more rare and *de novo* non-coding variants than can be reasonably followed up experimentally^6^, even with emerging high-throughput methods. We developed confidence scores to assess the support for a putative enhancer based on two criteria: (1) the statistical significance of the ChIP-seq enrichment observed at a locus and (2) the number of conditions under which a putative enhancer is observed (Methods section, Fig. 2b,c and Supplementary Data 2, Supplementary Note 3). For each predicted enhancer sequence we provide a combined score, on a scale from 0 (weakest evidence) to 1 (strongest evidence), reflecting the strength of evidence from all data sets combined. Enhancer usage has previously been shown to be temporally dynamic, with the majority of enhancers not active throughout the entirety of development^8^. Therefore, we have also provided scores from prenatal data alone and postnatal data alone. These stage-specific scores may be more applicable for studies, respectively, of congenital heart diseases, which typically present around birth, and phenotypes, such as coronary artery disease, that have adult onset.

We assessed the validity and utility of the confidence scores by comparing the genome-wide compendium against the collection of >2,000 experimentally tested enhancers available in the VISTA Enhancer Browser^24^. Many of the previously validated heart enhancers were retrospectively confirmed to be among the highest-ranking scored loci in the present study (Fig. 2d), and validation rates correlated positively with confidence scores (Fig. 2c, also see Supplementary Note 3, Supplementary Fig. 4, Methods section). In total, this retrospective analysis shows that the scoring scheme is a good indicator of the likelihood that a putative enhancer is active *in vivo*.

As an example of how this cardiac compendium can be integrated into human disease studies, we intersected it with variants associated with a variety of heart-related phenotypes reported in the NHGRI-EBI GWAS Catalog^28^. Including all variants in strong LD with the reported lead variants (*r*^2^≥0.8), more than 18,000 sequence variants are associated with human heart phenotypes. Approximately 2,300 of these fall within a predicted heart enhancer, with ∼900 in predicted enhancers that have a score of at least 0.2 (ranked list and corresponding heart enhancer scores provided in Supplementary Data 3). This includes a variety of loci where protein-altering variants have not been identified to explain the association signal, such as (1) the 6q22 region near *GJA1* that has been strongly implicated in heart rate^29^, (2) the 1p32 region overlapping *PLPP3* implicated in coronary artery disease^30^ and (3) the 15q24 locus containing *HCN4* implicated in atrial fibrillation^31^ (Supplementary Fig. 5). In the case of the *GJA1* locus, more than 200 single-nucleotide polymorphisms (SNPs) fall within multiple phenotype-associated LD blocks. Intersecting those SNPs with the heart enhancer compendium identified 11 variants that fall within predicted enhancers, including one in a very high-scoring element (score=0.719). This is a number that is tractable to downstream experimental validation. In the case of the *HCN4* locus, the strongest scoring putative enhancer in the region (score=0.313) includes a SNP (rs7172038) in perfect LD (*r*^2^=1) with the reported lead variant (rs7164883), and we included this enhancer in the functional validation performed below. Overall, the heart enhancer compendium can be easily intersected with human disease data to identify strong candidates for further experimental validation and should help prioritize enhancer sequence variants found in human resequencing studies.

### *In vivo* validation of heart enhancers near disease genes

**Figure 3:**
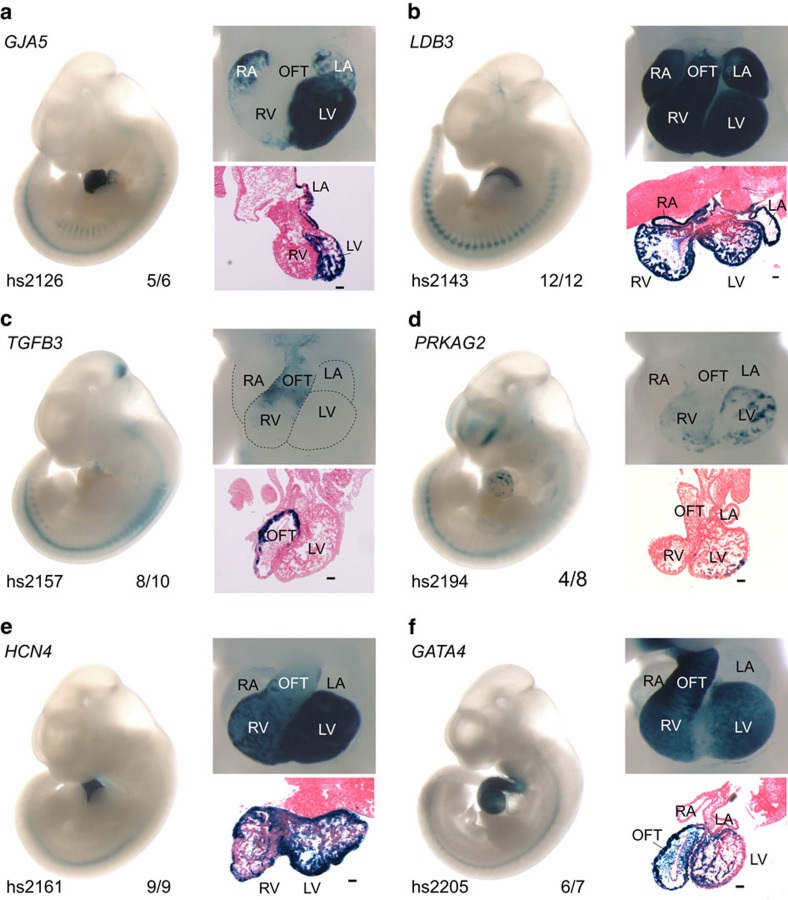
*In vivo* activity of human heart enhancers near heart disease genes. (**a**–**f**) Whole mount (left), heart close up (top right) and histological section (bottom right, scale bar, 100 μm) of E11.5 mouse embryos expressing a reporter *LacZ* gene (dark blue) under the control of a heart enhancer. VISTA Enhancer Browser identifier (hs number), nearest heart disease-associated gene, and the reproducibility of heart enhancer activity are indicated. LA, left atrium; LV, left ventricle; OFT, outflow tract; RA, right atrium; RV, right ventricle.

Given the large number of rare and *de novo* non-coding sequence variants that are identified in WGS studies, initial analyses will likely focus on characterizing variants in putative enhancers near genes previously implicated in heart disease. To explore the utility of the heart enhancer compendium for identifying *in vivo* enhancers near genes of interest, we examined 58 candidate sequences in transgenic mouse enhancer assays^32^,^33^. The selected regions spanned the full range of combined cardiac integrative analysis scores and were all located within 100 kb of a heart disease-associated gene (Supplementary Data 4). In addition to the close proximity, there is evidence from the chromatin–chromatin spatial interaction database^34^ that most of the tested sites physically interact with the putative target gene (Supplementary Data 4). In total, 22 of the tested sequences drove reproducible reporter gene expression in the developing heart or blood vessels at embryonic day 11.5 (E11.5) (Fig. 3, Supplementary Data 4 and Supplementary Table 6), including the enhancer upstream of *HCN4* that overlaps with a variant associated with atrial fibrilation. This enhancer had strong activity in the ventricles and weaker but reproducible activity in the atria (Fig. 3e). Examples of additional newly identified *in vivo-*validated cardiac enhancers include elements near *TGFB3* (Fig. 3c) and *PRKAG2* (Fig. 3d), two genes previously implicated in heart disease^35^,^36^ but without known associated distant-acting heart enhancers. Furthermore, we characterized new enhancers near genes such as *GATA4* (Fig. 3f, Supplementary Table 6), where several *in vivo* enhancers had been previously identified^15^,^37^. The overall transgenic validation rate (38% with cardiovascular activity) is somewhat lower than that reported by previous heart enhancer validation efforts. This is in large part due to our inclusion of candidate sequences from across the scoring range, in contrast to previous efforts, which tended toward validating loci with high ChIP-seq enrichment scores^14^,^15^. However, as expected, elements that were confirmed as *in vivo* cardiovascular enhancers had a higher average combined confidence score than those for which no cardiovascular activity was observed (mean combined scores: cardiovascular positive=0.509, cardiovascular negative=0.385, *P*=0.021, one-tailed *t*-test; Supplementary Data 4). These results illustrate the utility of the genome-wide scored data sets for identification of *in vivo* cardiac enhancers near heart disease genes of interest.

### Heart enhancer deletions result in cardiac dysfunction

**Figure 4:**
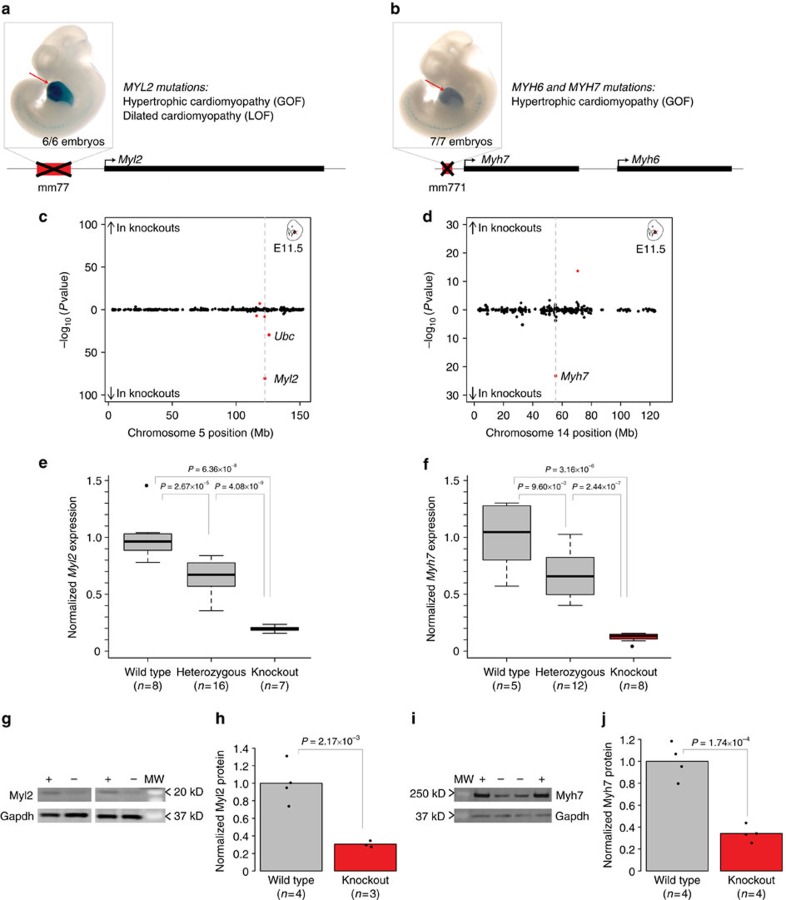
Cardiac enhancers are required for normal expression of heart disease-associated genes. Knockout analysis of enhancers mm77 (**a**,**c**,**e**,**g**,**h**) and mm771 (**b**,**d**,**f**,**i**,**j**). (**a**,**b**) Representative transgenic reporter assay results. Heart activity is indicated by red arrows. Numbers indicate reproducibility over total transgenic embryos. Gene models not drawn to scale. LOF: loss-of-function, GOF: gain-of-function. (**c**,**d**) Chromosome-wide mRNA expression changes in E11.5 whole heart. Points indicate individual genes, with red indicating statistically significantly differences after FDR correction (*P*<0.01 using an FDR<5%, see the ‘Methods' section for details). Dashed grey line indicates position of enhancer. Mb: megabases. (**e**,**f**) Normalized mRNA levels measured by quantitative RT-PCR in E11.5 hearts. Boxplots indicate median and quartile values for each data set; points indicate outliers. (**g**–**j**) Representative western blot images (**g**,**i**) and normalized protein levels (**h**,**j**) in wild-type (+) and homozygous null (−) samples. Gapdh was used as a loading control, MW, molecular weight. (**g**,**i**). Bars indicate group means, and points represent biological replicates (**h**,**j**). For (**e**,**f**,**h**,**j**): values were normalized to the wild-type mean, and *P* values were calculated using a one-tailed *t*-test.

While *in vivo* reporter assay validation is a powerful tool to confirm that an enhancer is sufficient to activate tissue-specific gene expression, it does not illuminate whether a sequence is necessary for proper development or health. Such information is, however, crucial to understanding the phenotypic consequences of non-coding mutations in the human genome. To assess the biological necessity of heart enhancers for proper cardiac development and function, we created knockout mouse models for two different heart enhancers, mm77 and mm771, upstream of *Myl2* and *Myh7*, respectively. Mutations in either of these genes are associated with hypertrophic cardiomyopathy, and their coding sequences are routinely screened for mutations in the clinic^4^. Both enhancers fall in regions with very high combined confidence scores (0.828 and 0.756, respectively). Furthermore, both drive strong, reproducible reporter gene expression throughout the heart at E11.5 and were initially identified from epigenomic data sets considered in the present integrative analysis (Fig. 4a,b, Supplementary Fig. 6). In both cases, the human and mouse orthologous enhancer sequences drive strong and highly reproducible reporter activity in the heart in mouse transgenic assays, indicating functional conservation of these enhancers in mammals (Supplementary Fig. 6).

We created two mouse lines, each carrying a deletion of one of these two enhancers (Supplementary Fig. 7). Mice homozygous for either enhancer deletion are born at normal Mendelian ratios and show no gross abnormalities or overt impairments of health (Supplementary Table 7). Adult females heterozygous or homozygous null for mm771 have 5–10% lower body weight than wild-type females (*P*<0.05, one-tailed *t*-test), but no growth phenotypes were observed for mm771 males or for mm77-null mice of either gender (Supplementary Fig. 8).

To assess gene expression changes resulting from the loss of either enhancer, we performed RNA sequencing on heart tissue from E11.5 and adult mice from each enhancer deletion line. In mice homozygous null for mm77, *Myl2* RNA expression is reduced by 75–80% compared with wild-type levels at both E11.5 and in adulthood (both *P*<4.9 × 10^−19^, see the ‘Methods' section, Fig. 4c, Supplementary Fig. 9, Supplementary Data 5), establishing that *Myl2* is a direct regulatory target and that the enhancer is required for normal expression of *Myl2* in both embryonic and adult heart. In addition to *Myl2*, embryonic and adult mm77-null animals show a deficit of *Ubc*, a ubiquitin gene more than 3 Mb away from the enhancer (both *P*<5.3 × 10^−11^). Embryonic *Δmm771* mice show downregulation of *Myh7* by ∼85% compared with wild-type (*P*=5.7 × 10^−24^) but show no change in *Myh6* or any other gene in *cis*, indicating that the enhancer specifically controls the expression of *Myh7* (Fig. 4d, Supplementary Data 5). *Myh7* is not expressed in postnatal mouse heart^38^, and, consequently, no significant changes to its expression were observed in adult *Δmm771* mice (Supplementary Fig. 10a, Supplementary Data 5). In all cases, the expression changes observed by RNA-seq were confirmed by targeted quantitative reverse transcriptase PCR (qRT-PCR; Fig. 4e,f, Supplementary Figs 9c and 10b). For the affected myosin genes, gene expression changes are dose dependent, with heterozygotes having a reduction in myosin gene expression by ∼35% compared with wild-type (mm77: *P*=2.7 × 10^−5^, mm771: *P*=9.6 × 10^−3^, one-tailed *t*-test, Fig. 4e,f). We next examined whether these mRNA expression changes result in decreased myosin protein abundance. Western blot analysis on whole-heart tissue established that both enhancer deletions result in ∼70% reduction of cardiac protein levels (Myl2, *P*=2.1 × 10^−3^; Myh7, *P*=1.7 × 10^−4^, one-tailed *t*-test; Fig. 4g–j, Supplementary Fig. 11). Cumulatively, these results show that mm77 and mm771 are required for normal levels of Myl2 and Myh7 protein in the heart.

**Figure 5:**
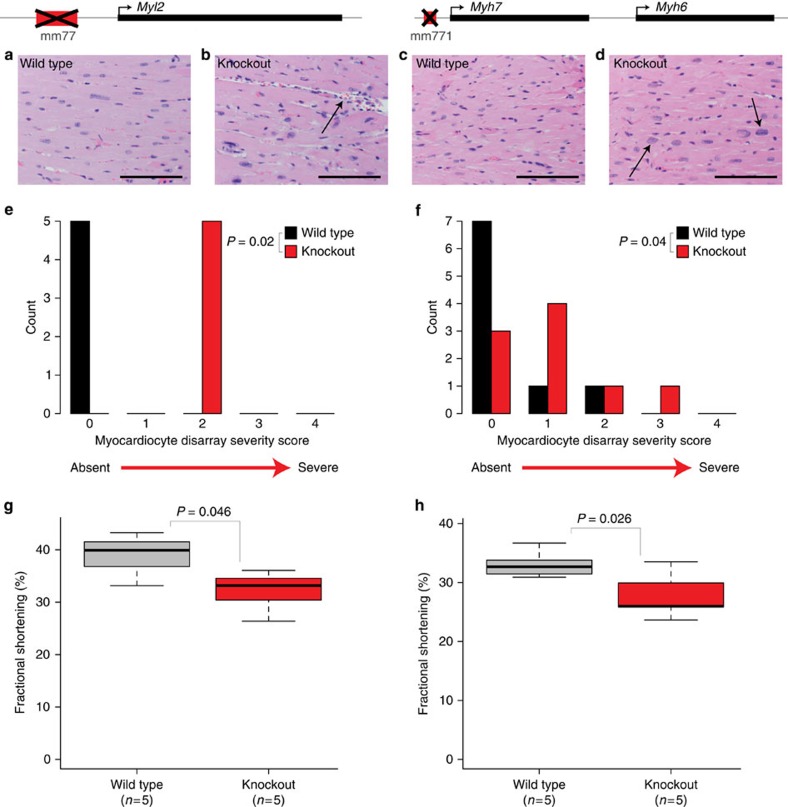
Loss of heart enhancers results in cardiac abnormalities. (**a**–**d**) Representative images of haematoxylin and eosin stained heart tissue from mice wild-type (**a**,**c**) or homozygous null (**b**,**d**) for the mm77 (**a**,**b**) or mm771 (**c**,**d**) enhancers. (**b**) Characteristic myocardiocyte disarray (arrow) observed in a homozygous *Δmm77* mouse. (**d**) Myocardiocyte karyomegaly (arrows) observed in a homozygous *Δmm771* mouse. Scale bars, 100 μm. (**e**,**f**) Severity of myocardiocyte disarray observed in the hearts of mice wild-type or homozygous null for the mm77 (**e**) or mm771 (**f**) enhancer. Cardiac tissue was scored by a genotype-blind pathologist from 0 (absent) to 4 (severe), and *P* values calculated by one-tailed paired Wilcoxon rank-sum test. (**g**,**h**) Cardiac fractional shortening for mice wild-type and homozygous null (knockout) for the mm77 (**g**) or mm771 (**h**) enhancer. For (**g**,**h**) Boxplots indicate median, range and quartile values for each data set, and *P* values were calculated by one-tailed paired *t*-test.

To evaluate potential cardiac dysfunction resulting from the loss of the *Myl2* or *Myh7* enhancer, we performed histological and pathological analysis on both lines (Supplementary Data 6). In heart tissue from both *Δmm77* and *Δmm771* mice, we observed cases of myocardiocyte disarray and karyomegaly (cell nucleus enlargement; Fig. 5a–d). To quantify these observations, we used histological severity scores assigned by a genotype-blind pathologist. Compared with wild-type littermates, we observed substantially increased rates and severity of myocardiocyte disarray both in *Δmm77* (*P*=0.02, paired one-tailed *t*-test) and *Δmm771* (*P*=0.04) mice, as well as karyomegaly in *Δmm77* (*P*=0.02, Fig. 5e,f, Supplementary Fig. 12). In contrast, no reproducible histological or pathological abnormalities were observed in any other major organ system (Supplementary Data 6). These results support that the deleted enhancers are important for establishing appropriate levels of myosin gene products and for maintaining healthy cellular morphology in the heart.

To further assess the effect of heart enhancer loss on cardiac physiological function, we used echocardiography. Homozygous *Δmm77* (*P*=0.046, paired one-tailed *t*-test) and *Δmm771* (*P*=0.026) mice had modest but significant decreases in fractional shortening (Fig. 5g,h, Supplementary Fig. 13a,b), as well as decreases in ejection fraction for *Δmm771* (*P*=0.025, Supplementary Fig. 13c–f). These results are consistent with an early-stage cardiomyopathy phenotype, specifically dilated cardiomyopathy (Supplementary Note 4, Supplementary Fig. 14). This conclusion is further supported by adult *Δmm77* mice showing upregulation of *Nppa* (RNA-seq: *P*=8.29 × 10^−10^, see the ‘Methods' section) and *Nppb* (RNA-seq: *P*=2.59 × 10^−07^) (Supplementary Figs 9b and 15), genes whose upregulation is a common biomarker of heart failure^39^. Overall, the loss of either of the cardiac enhancers results in a large decrease of the mRNA and protein products of a neighbouring cardiac myosin gene, which leads to cardiac cellular abnormalities and reduced heart function. These results illuminate a potential role for enhancer mutations in human heart disease.

## Discussion

Technological advances have enabled routine WGS for the study of human disease. However, initial WGS analyses have focused primarily, or exclusively, on the <2% of the genome that encodes proteins^6^,^40^. While the strong contribution of non-coding sequences in many human disease traits is now widely recognized^41^, the skew towards coding sequence in the analysis of WGS data persists because of a lack of annotations and analytical tools to assess whether rare non-coding mutations are associated with phenotypes^6^. In the present study, we begin to fill this void by generating a comprehensive compendium of more than 80,000 enhancers predicted to be active in the developing and adult human heart. Since these sequences represent functional units with defined boundaries, this allows for the binning of rare non-coding variants for association testing, analogous to aggregating coding variants by gene^42^. To facilitate the prioritization of non-coding regions for experimental follow-up studies, each predicted enhancer is provided along with a confidence score summarizing the strength of the supporting epigenomic evidence. While the present study focuses on a single organ system of major epidemiological importance, the approach described here is applicable to nearly all human organ systems. Thus, we expect that similar resources for other phenotype-relevant tissues will serve as a critical foundation for the analysis of genetic data from many additional classes of human disease.

Non-coding sequence annotation is a critical first step for interpreting whole-genome sequence data but is generally insufficient to conclusively implicate specific mutations as causal in disease. One powerful complementary approach is through the use of genome engineering in animal models to formally test the importance of a sequence change *in vivo*. In this study, we illustrate how enhancers identified using genome-wide approaches can be prioritized and assessed for normal organismal function. Focusing on two heart enhancers located near disease-associated genes, we show in mouse models how their loss of function results in cardiac phenotypes. While a few enhancers previously knocked-out in mice have the potential to result in cardiac phenotypes (for example, refs 43, 44), to our knowledge, the *Myl2* and *Myh7* enhancers are the first examples whose loss has been shown to result in a phenotype consistent with heart disease. With the recent advances in genome editing technology^45^, it appears likely that *in vivo* engineering approaches will be increasingly used for large-scale modelling of both coding and non-coding human mutations. Overall, this study highlights the important role enhancers play in cardiac health and provides a valuable compendium of human heart enhancers that can be easily integrated into cardiovascular disease studies.

## Methods

### Chip-seq data analyses for heart enhancer prediction

Short read data of published data sets were retrieved from the Gene Expression Omnibus^46^ or Human Roadmap Epigenome website (http://www.ncbi.nlm.nih.gov/geo/roadmap/epigenomics/). Unpublished ENCODE data was accessed through the ENCODE Data Coordination Center website (https://www.encodeproject.org/search/). Supplementary Table 1 lists details about each sample included in this meta-analysis. In the case of reads stored in the Short Read Archive format^47^, fastq files were obtained using the *fastq-dump* script available in the sratoolkit (v2.4.5).

Alignments to either the mouse (mm10) or human (hg19) reference genomes were performed using Bowtie v0.12.7 (ref. 48). Only reads with a unique match to the genome and showing two or fewer mismatches (-m 1 -v 2) were retained. Peak calling was performed using MACS v1.4 (ref. 49) with the following parameters: *-gsize=mm -bw=300 -nomodel -shiftsize=100*. Whenever available, the experiment-matched input DNA was used as control. When this was not available (Supplementary Table 1), peaks were called without using an input DNA as control with MACS v1.4 parameter* -nolambda*.

The resulting lists of peaks were annotated to the nearest RefSeq (ref. 50) gene TSS in either mm10 or hg19 using HOMER^51^. Enriched regions with their annotated centre within 1.5 kb from any TSS were considered promoters (Supplementary Data 1) and separated from the putative enhancers for the scoring described below. In case of very large regions of enrichment, the centre might happen to lie outside the ±1.5 kb from annotated TSSs. To avoid considering them, *subtractBed* (ref. 52) was used to avoid any overlapping sub-interval. mm10 v3 and hg19 v19 Basic Gencode annotations^53^ were downloaded from the UCSC Genome Browser^54^ on 3 June and 12 July 2015, respectively. Filtering for the TSS-proximal regions of Gencode annotated transcripts also ensured the exclusion of potential promoters of non-coding genes from the final list.

In some cases, multiple H3K27ac data sets were available from independent sources for the same mouse developmental time point, and these samples were treated as biological replicates. Biological replicates were combined using MSPC (ref. 55), using the following parameters *-r biological -s 1E-10 -W 1E-6*. The confirmed peaks were assigned the best *P* value, as defined by MACS, among the overlapping peaks.

### Meta-analysis and annotation of heart enhancers

The obtained lists of putative enhancer regions for the mouse samples (mm10) were mapped to the human genome (hg19) using liftOver^54^ with a requirement that ≥50% of the bases in each region map to human. Beside, each region was required to both uniquely map to hg19, and to uniquely map back to the original region in mm10 (similar to ref. 56). Human, as well as human-lifted mouse, regions were then merged together using *mergeBed*^52^. The resulting regions were re-annotated to the lowest *P* value among those shown by the overlapping regions; only those regions showing at least one peak with a *P* value ≤1e-10 (as defined by MACS) in at least one condition were considered for further analysis.

To exclude potential technical artifacts, published H3K27ac data sets from human cell lines^57^ were used to produce a blacklist of regions systematically enriched across all ChIP-seq data sets. A blacklist of human regions generated by the ENCODE Consortium itself was also used^57^ (https://sites.google.com/site/anshulkundaje/projects/blacklists).

Gene ontology enrichment was performed for the complete list of putative heart enhancers using GREAT^23^.

The positions of the resulting regions were annotated relative to the TSS of: (1) the nearest gene; (2) the nearest gene showing one or more heart-related phenotypes in the Mouse Genome Database^58^; and (3) the nearest gene annotated with a heart-related phenotype in the Human-Phenotype Ontology^59^. These annotations were performed using a custom script and lists of RefSeq genes downloaded from the UCSC Genome Browser 4 May 2015. The manually curated lists of heart-related terms are included in Supplementary Data 7. The putative enhancer regions were also annotated to variants significantly associated to human phenotypic traits—as listed in the GWAS catalogue^28^. *Bedmap* from the BEDOPS suite^60^ was used to perform this annotation step.

### Scoring scheme for putative heart enhancers

Given one condition (for example, E11.5 mouse H3K27ac), for each given putative enhancer region *r* and its MACS-generated *P* value *p*, the following score *S* was calculated:


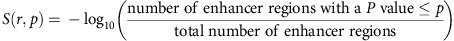


Each score represents the probability of observing an equal or better ChIP-seq enrichment (that is, an equal or lower *P* value), under a specific condition. For each region, the scores were summed up either across all conditions (to obtain the final combined score) or only across foetal or postnatal conditions (to get the pre- and postnatal scores, respectively). Scores were normalized on a scale of 0.0–1.0 such that the highest scoring enhancer in each score class (combined, prenatal and postnatal) was set to 1.0.

### Intersecting enhancer predictions with VISTA

Regions experimentally tested for *in vivo* enhancer activity in mouse transgenic assays were downloaded from the VISTA enhancer browser (http://enhancer.lbl.gov/)^24^ on 24 September 2015. Only VISTA elements that could be mapped to hg19 were considered, and those that overlapped promoters or blacklisted sequences (as defined in the meta-analysis section above) were excluded. The overlap among VISTA elements and the putative heart enhancers resulting from the meta-analysis was assessed using *coverageBed*^52^. Only regions and VISTA elements intersecting at least 500 bps were considered for further analyses. The overlapping VISTA elements (both positive and negative for enhancer activity) were then ranked based on the score, and a cubic spline (df=3) was fit using the *splineFit* function available in R. This way, a curve estimating the value of *in vivo* validation rate across the whole spectrum of scores over the validated elements was obtained. These values were then used to extrapolate the curve that directly relates the *in vivo* validation rate to the score of all regions in the meta-analysis. This procedure was repeated separately for each of the three scores.

### Comparison with other enhancer prediction methods

We compared our unsupervised enhancer prediction against two previously reported methods, EnhancerFinder^27^ and EMERGE^26^. Unless otherwise noted (Supplementary Table 5), area under the curve for receiver operating characteristic curves for EnhancerFinder and EMERGE results are those reported in the corresponding papers for the prediction of mammalian heart enhancers. All other area under the curve values were generated as described in the EMERGE paper^26^.

### Intersecting heart enhancer compendium with GWAS catalogue

We retrieved all phenotype-associated SNPs in the NHGRI-EBI GWAS catalogue^28^ implicated in heart-related traits (search term ‘heart'). The SNP Annotation and Proxy Search (SNAP)^61^ tool was used to identify all SNPs in strong LD using the following parameters: 1,000 Genomes Pilot 1 data set, *r*^2^≥0.8, the Northern Europeans from Utah (CEU) population panel, 500 kb distance limit.

### Transgenic mouse assays

Enhancer and allelic variant names (mm and hs numbers) used in this study are the unique identifiers used in the VISTA Enhancer Browser (http://enhancer.lbl.gov/). Enhancer sequences were amplified from human (hs numbers) or mouse (mm numbers) genomic DNA and cloned into an hsp68-lacZ expression vector as previously described^33^. Genome coordinates and primer sequences for all elements are listed in Supplementary Data 4 and Supplementary Table 8. Transgenic mouse assays were performed as previously described^32^,^33^, and results for mm77 (ref. 15) and the human ortholog of mm771 (hs1670)^62^,^63^ were previously reported. To determine if tested enhancers physically contact the putative target genes, we queried all available human and mouse data sets in the chromatin–chromatin spatial interaction database^34^. To avoid considering interactions that could have been annotated just due to physical proximity, enhancer-promoter pairs closer than 10 kb were not annotated (indicated as ‘Close' in Supplementary Data 4).

### Generation of enhancer knockout mice

Enhancer null lines were generated via homologous recombination (Supplementary Fig. 7a) as previously described^62^. Primer sequences used for generating, validating (Supplementary Fig. 7b,c) and genotyping (Supplementary Fig. 7d,e) targeted ES cell lines and mice are listed in Supplementary Table 9. The mm771 deletion removed 332 bp (mm10 chr14:54,996,893–54,997,224) and the mm77 deletion removed 2,517 bp (mm10 chr5:122,092,252–122,094,768) of non-coding sequence.

### Gene expression analysis for enhancer knockouts

RNA was isolated from whole hearts microdissected from multiple litters of embryonic mice using the Ambion RNAqueous Total RNA Isolation Kit (Life Technologies) according to manufacturer instructions. Matched genomic DNA was collected for each embryo from limb or tail tissue as previously described^62^. Each embryo's enhancer genotype was assessed using the mm77 or mm771 genotyping primers listed in Supplementary Table 9. For adult mice, RNA was extracted from the ventricular portion of the heart using physical homogenization and the TRIzol Reagent (Life Technologies).

For RNA-seq, RNA samples were DNase-treated with the TURBO DNA-free Kit (Life Technologies), and RNA quality was then assessed using a 2100 Bioanalyzer (Agilent) with an RNA 6,000 Nano Kit (Agilent). RNA sequencing libraries were made using the TruSeq Stranded Total RNA with Ribo-Zero Human/Mouse/Rat kit (Illumina) or the TruSeq Stranded mRNA Sample Prep Kit (Illumina) according to manufacturer instructions. RNA-seq libraries were subjected to an additional purification to remove remaining high molecular weight products as follows: sample volume was increased to 100 μl by addition of 1X TE buffer or Illumina Resuspension Buffer and then incubated with 60 μl Agencourt AMPure XP beads for 4 min. The beads were pelleted by incubation on a magnet, and the entire supernatant was transferred to a tube containing 50 μl of fresh AMPure XP beads and incubated for 4 min. After pelleting the new beads with a magnet, the supernatant was discarded, the beads washed twice with 80% ethanol and the DNA was eluted in 30 μl Illumina Resuspension buffer. The resulting RNAseq libraries were diluted 10 × and their quality and concentration were assessed using a 2100 Bioanalyzer with the High Sensitivity DNA Kit (Agilent) and a Qubit Fluorometer with the Qubit dsDNA HS Assay Kit (Life Technologies). RNAseq libraries were pooled four libraries per lane and sequenced via single end 50 bp reads on a HiSeq 2000 (Illumina).

RNA-seq data was analysed as follows: CASAVA v1.8.0 (Illumina) was used to demultiplex data, and reads with CASAVA ‘Y' flag (purity filtering) were discarded. After quality filtering and adaptor trimming using cutadapt_v1.1 (ref. 64) with parameter ‘-m 25 -q 25', between 37 and 54 million reads were obtained for each sample. Mouse genome sequence (mm9) and gene annotation were retrieved from the iGenomes repository (https://support.illumina.com/sequencing/sequencing_software/igenome.html). Tophat v2.0.6 (ref. 65) was used to align the reads to the mouse reference genome and transcriptome, then reads mapping to UCSC known genes were counted by HTSeq (ref. 66). Differential gene expression analysis between wild type and knockout for mm77 or mm771 enhancers was performed using edgeR (ref. 67), and genes whose expression was extremely low in all samples (fragments per kilobase of transcript per million mapped reads (FPKM)<1, calculated by Cufflinks v2.2.1 (ref. 68)) were discarded for further analysis. *P* values for all RNA-seq experiments are those reported by edgeR after false discovery rate (FDR) correction (FDR<5%).

For quantitative RT-PCR (qPCR) measuring *Myl2*, *Myh7* and *Ubc*, RNA was reverse transcribed using SuperScript III (Life Technologies) with random hexamer or poly-dT priming according to manufacturer instructions. For qPCR measuring *Nppa* and *Nppb*, RNA was first treated with RNase-free DNase (Promega) and then reverse transcribed using SuperScript III with poly-dT priming. qPCR was performed on a LightCycler 480 (Roche) using TaqMan-style reactions containing Master Mix (LightCycler 480 Probes Master Mix (Roche) or Probe Fast Universal qPCR Master Mix (Kapa Biosystems)), PrimeTime qPCR Assay primer/probe mix (Integrated DNA Technologies) for the test gene, primer/probe mix to the actin control gene, 0.5–1 μl of the reverse transcriptase reaction and RNAase-free water. qPCR assays were performed in triplicate for each sample, and genomic DNA amplification was excluded for all samples by a lack of substantial amplification in reverse transcriptase-negative qPCR reactions or by the absence of a genomic DNA amplification band when reverse transcriptase-positive reactions were run on an agarose gel. PrimeTime qPCR Assay primer/probe mixes used for qPCR assays are provided in Supplementary Table 10. Reactions were analysed as previously described^69^, using actin as the reference gene.

### Protein expression analysis for enhancer knockouts

Myosin was extracted from individual embryonic or adult mouse whole hearts as previously described^70^ with minor modifications. Protein was quantified by Bradford Assay. Proteins were separated on 8–16% gradient Tris-glycine gels by electrophoresis and transferred to polyvinylidene difluoride membranes using standard western blotting procedures. Membranes were blocked with 5% bovine serum albumin, incubated with primary antibodies overnight, washed, incubated with secondary antibodies for 1 h, washed and imaged using a VersaDoc Molecular Imager (Bio-Rad). Antibody details are provided in Supplementary Table 11. Quantification of band intensities was carried out using the gel analyzer feature of ImageJ (ref. 71), with GAPDH serving as a loading control. Uncropped western blot images are provided in Supplementary Fig. 11.

### Echocardiography, necropsy, pathology and histology

Echocardiography was performed by the University of California Davis Mouse Metabolic Phenotyping Center's Cardiovascular Biology and Pathology Core. Echocardiograms were performed on conscious animals to assess the systolic function using M-mode and two-dimensional measurements as previously described^72^.

Gross necropsy, histology and pathology were performed by the University of California Davis Comparative Pathology Lab using standard techniques. Briefly, animals were euthanized by carbon dioxide asphyxiation. Organs were collected, weighed and fixed with paraformaldehyde. Tissues were paraffin embedded, and 5 μm sections were stained with haematoxylin and eosin for pathological evaluation.

Cardiac histology sections were assessed by a trained veterinary pathologist, who was blinded to genotype status. The severity of left ventricular hypertrophy, myocardiocyte disarray, myocardiocyte karyomegaly and interstitial fibrosis present in each heart sample were scored as follows:

0=not observed

1=minimal (1–2 foci or <10% of heart involved)

2=mild (3–6 foci or 10–40% of heart involved)

3=moderate (6–10 foci or 40–60% of heart involved)

4=severe (10+ foci or >60% of heart involved)

### Animal approval and experimental design

All animal work was reviewed and approved by the Lawrence Berkeley National Laboratory Animal Welfare and Research Committee or the University of California, Davis Institutional Animal Care and Use Committee.

R was used to compute the statistics and to generate plots for this work.

#### Transgenic mouse assays

Transgenic assays were performed in *Mus musculus* FVB strain mice. Sample sizes were selected empirically based on our previous experience of performing transgenic mouse assays for >2,000 total putative enhancers (for example, refs 12, 14, 15, 33, 62). Mouse embryos were only excluded from further analysis if they did not carry the reporter transgene or if they were not at the correct developmental stage. As all transgenic mice were treated with identical experimental conditions, and as there were no groups of animals directly compared in this section of the study, randomization and experimenter blinding were unnecessary and not performed.

#### Enhancer knockouts

Enhancers were deleted in *Mus musculus* W4 (129S6 strain) mouse embryonic stem cells (Taconic). Resulting mice were crossed into the C57BL/6J strain. All mice and mouse embryos described in the enhancer knockout section of this paper resulted from heterozygous x heterozygous crosses to allow for the comparison of matched littermates of different genotypes. With the exception of qPCR experiments (where entire litters were analysed) and the RNA-seq performed on adult mm77 mice (where sufficient matched littermates were not available), all experiments employed a matched littermate selection strategy. For every homozygous null animal selected, a homozygous wild-type animal from the same litter was selected for comparison. For all postnatal mice, littermate pairs were selected to have matching genders. Embryonic samples used for qPCR, RNA-seq and western blotting were dissected blind to genotype.

The underlying hypothesis of the enhancer knockout section was that loss of either cardiac enhancer would decrease neighbouring myosin gene and protein levels. Based on the *Myl2* gene-null phenotype previously observed in humans and mice (see Supplementary Note 4), we expected this decrease in gene expression to result in reduced cardiac function and possibly mild cardiac hypertrophy in homozygous null mice relative to matched wild-type littermates. Therefore, statistical significance of results was assessed by one-tailed *t*-test or Wilcoxon rank-sum test for metrics such as gene/protein expression, heart weight/body weight ratios, left ventricular mass and cardiac function (ejection fraction and fractional shortening) comparisons. Remaining metrics were assessed using two-tailed tests. Because of the matched littermate selection scheme used, paired tests were used to assess significance of echocardiography and pathology results.

#### qPCR

Embryonic samples were collected from at least two independent litters for each line. Embryos were excluded from any further analysis if they were in the process of being resorbed, not at the correct developmental stage, or insufficient quantities of RNA were isolated from them. Otherwise, all embryonic samples collected for each qPCR experiment were analysed. Adult samples for qPCR validation of *Nppa*, *Nppb* and *Ubc* were technical replicates of those used for RNA-seq.

#### RNA-seq

Samples were chosen for RNA-seq based on RNA sample quantity and quality, and the availability of matched littermate pairs. To avoid batch effects, RNA-seq libraries from all samples within the same experiment were made in the same batch. Libraries were pooled together such that matched littermate pairs were run on the same flow cell lane.

#### Western blots

One potential homozygous null mm77 animal was excluded from analysis due to inconclusive genotyping results, resulting in one unmatched wild-type sample in this analysis. To avoid batch effects, homozygous null and homozygous wild-type samples were alternated for protein extraction, and all samples within an experiment were run on the same gel and blot.

Echocardiography, Histology and Pathology: to control for any physiological effects due to strain background, age or gender, littermate pairs were selected for further phenotyping such that one mouse in each pair was homozygous wild-type and one mouse was homozygous null and both mice in each pair were the same gender. To minimize physiological effects due to body weight, littermate pairs were selected to minimize differences in body weights between paired samples. Detailed information (age, sex, body mass and so on) about mice selected for this phenotyping is provided in Supplementary Data 6. All echocardiography, necropsy, pathology and histology assays were performed blinded to genotype, and mice were randomized for echocardiography. Sample sizes were selected for echocardiography based on similar previously reported studies^73^. Left ventricular mass could not be ascertained by echocardiography for one mouse homozygous null for the mm771 enhancer.

### Data availability

RNA-seq files are available in the NCBI GEO database with the accession code GSE75907. All other data that support the findings of this study are available from the corresponding author upon request.

## Additional information

**How to cite this article:** Dickel, D. E. *et al*. Genome-wide compendium and functional assessment of *in vivo* heart enhancers. *Nat. Commun.* **7,** 12923 doi: 10.1038/ncomms12923 (2016).

## Supplementary Material

Supplementary Information Supplementary Figures 1-15, Supplementary Tables 1-11. Supplementary Notes 1-4 and Supplementary References

Supplementary Data 1 Human promoters active in the heart identified by integrative analysis. H3K27ac and p300 peaks within 1.5 kb of a gene transcription start site were considered to be promoters. Genome coordinates (chromosome, start, end, size) are for the hg19 human reference genome. Integrative analysis scores (0-1) are provided for All heart samples, and just Prenatal or Postnatal samples. For each promoter region, we have indicated the name of the Gencode or RefSeqannotated gene, along with the distance between its transcription start site and the center of the H3K27ac/p300-defined region. The "GWAS" column indicates any SNPs, and their associated human phenotype(s), that fall into the H3K27ac/p300-defined promoter regions.

Supplementary Data 2 Putative human heart enhancers identified by integrative analysis. Human (hg19) coordinates (chrom, start, end, size) are given for each putative heart enhancer identified. For each locus, a normalized confidence score (0-1) is provided indicating the support that a site is a cardiac enhancer 1) at any time (score_All), 2) during prenatal development (score_Prenatal), or 3) in postnatal development (score_Postnatal). For each locus, we include 1) the nearest gene and 2) the nearest genes with a heart-related phenotype as ascertained in mouse (MGI) or human (HPO). The distance to nearest gene is given in bases from the enhancer to the gene's transcription start site. The VISTA columns indicate whether the locus overlaps any sites that have been tested in transgenic mice and deposited in the VISTA enhancer database: 1) VISTA_positive: enhancer activity found in any tissue; 2) VISTA_negative: no reproducible enhancer activity in any tissue; and 3) VISTA_positive_heart: reproducible enhancer activity observed in heart. The GWAS column lists variants in each putative enhancer that are associated with the indicated human phenotype(s). For "Overlap Gene Body", "VISTA_positive", "VISTA_negative", and "VISTA_positive_heart", the numbers (0-2) indicate the number of genes or VISTA elements overlapping the scored region. Note that seven VISTA_positive_heart elements overlap two scored regions each.

Supplementary Data 3 Heart phenotype-associated SNPs in putative heart enhancers. Heart phenotype-associated variants were identified from the NHGRI-EBI GWAS Catalog (search term: "heart") and expanded to include all variants in strong LD (r2 >=0.8) with the reported SNP. Of the >18,000 heart phenotype-associated SNPs, ~2,300 fall into putative heart enhancer regions. Genome coordinates for the associated SNPs and their corresponding enhancers are given in hg19. The list is sorted in descending order of enhancer score (Score_All).

Supplementary Data 4 Transgenic assays for putative human heart enhancers near heart disease genes. The human homolog of each locus was tested for enhancer activity in embryonic day 11.5 transgenic mice. Name indicates the VISTA identifier. Genomic locations and scores are given as in Supplementary Data 2. "Nearest HD Gene" indicates the closest heart disease-associated gene. "Distance to HD Gene" indicates the distance between the physical center of the enhancer locus and the closest annotated transcription start site in Gencode for the listed HD gene, in both the human (hg19) and mouse (mm10) genomes. "Interaction with Nearest HD Gene" indicates whether there was evidence for a physical interaction between the tested enhancer and the putative target gene in the Chromatin-Chromatin Spatial Interaction (CCSI) database for either human or mouse samples ("Close" indicates that distance between the enhancer and promoter is below the ~10 kb resolution of the chromatin conformation results). Forward and Reverse Primers indicate the primers used to amplify each site. "Transgenic Result" indicates tissues (or lack thereof) where reproducible enhancer activity was observed at E11.5.

Supplementary Data 5 Differentially expressed genes in enhancer deletion lines. All genes that were differentially expressed (P-value < 0.01 using an FDR < 5%) in embryonic or adult heart from mm77 and mm771 knockout lines, as ascertained by edgeR. "logKOWT" indicates log (mean knockout expression over mean wild-type expression). Negative values indicate genes downregulated in the knockout animals. "logCPM" indicates log(mean expression level over all samples in counts per million). Genes in bold italics are highlighted in the main text.

Supplementary Data 6 Histology and pathology results for enhancer knockout mice. * WT: homozygous wild-type, KO: homozygous null; ** NSF: no significant finding, n/a: detailed comment or value not recorded.

Supplementary Data 7 List of heart related gene terms considered from the Mouse Genome Database (MGD) and the Human Phenotype Ontology (HPO)

## Figures and Tables

**Figure 1:**
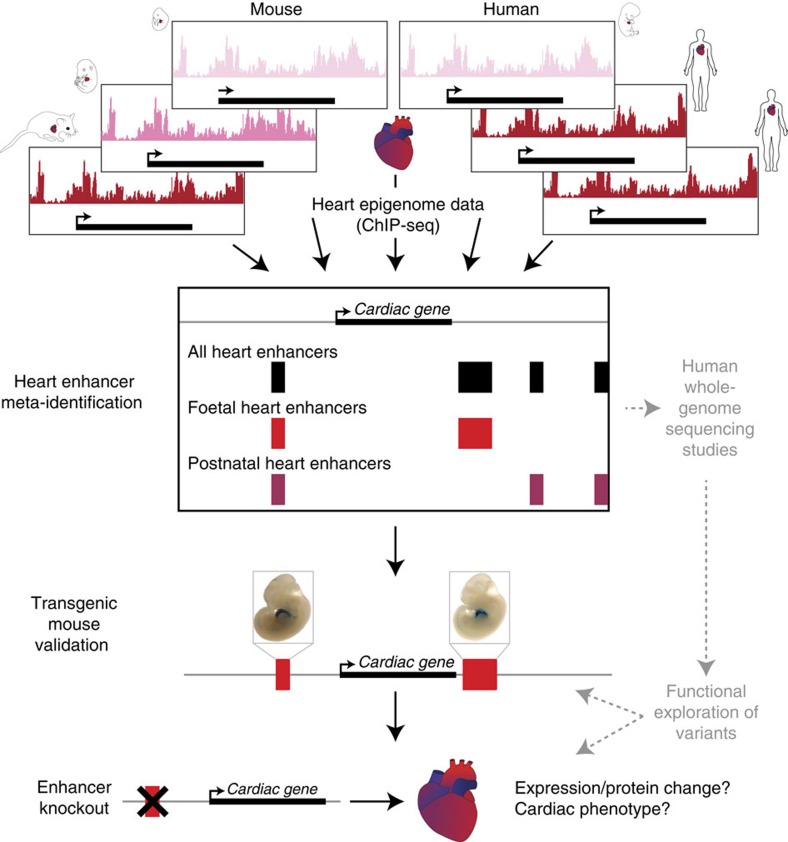
Generation and validation of a genome-wide cardiac enhancer catalogue. Integrative analysis of >35 epigenomic data sets from *ex vivo* human and mouse heart tissue resulted in a catalogue of >80,000 putative human heart enhancers. We demonstrate the utility of this catalogue for the discovery of enhancers near heart disease-associated genes by characterizing the *in vivo* activity patterns of 22 novel cardiovascular enhancers in transgenic mouse assays. We also show the functional importance of enhancers by deleting two cardiac enhancers, which resulted in reduced gene expression and impaired cardiac function.

**Table 1:** Data sets included in the integrative epigenomic analysis.

Species/stage/anatomical region	Enhancer mark	References
*Human*		
Prenatal (16 week gestation), whole heart	p300/CBP	14
3 years, left ventricle	H3K27ac	16
3 years, right ventricle	H3K27ac	16
30 years, aorta	H3K27ac	16
34 years, aorta	H3K27ac	16
34 years, left ventricle	H3K27ac	16
34 years, right ventricle	H3K27ac	16
34 years, right atrium	H3K27ac	16
45 years, septum	p300/CBP	14
*Mouse*		
E11.5, whole heart	H3K27ac, p300	8,15 Unpublished (ENCODE)
E13.5, whole heart	H3K27ac	Unpublished (ENCODE)
E14.5, whole heart	H3K27ac	8,17,18
E15.5, whole heart	H3K27ac	Unpublished (ENCODE)
E16.5, whole heart	H3K27ac	Unpublished (ENCODE)
E17.5, whole heart	H3K27ac	8
P0, whole heart	H3K27ac	8, Unpublished (ENCODE)
P5, whole heart	p300	19
P7, whole heart	H3K27ac	8
P21, whole heart	H3K27ac	8
P56, whole heart	H3K27ac, p300	8,17,18

Epigenomic profiles for enhancer-associated marks (H3K27ac and p300/CBP) are from human and mouse heart tissue at a variety of developmental stages. ENCODE indicates previously unpublished data from the ENCylopedia of DNA Elements project. Developmental stages for mouse are given in embryonic days post-conception (E) or days after birth (P). With the exception of the human septum sample, all tissue samples were reported to be normal, or no explicit statement of health was provided in the referenced publication.

**Table 2:** Top enriched human phenotypes of putative target genes near predicted heart enhancers.

Top enriched phenotypes	Binomial FDR *Q*-value	Binomial fold enrichment
Cardiac arrest	6.81 × 10^−89^	2.0
Sudden cardiac death	7.84 × 10^−89^	2.0
Sudden death	3.67 × 10^−84^	3.1
Syncope	1.22 × 10^−64^	2.2
Atrial fibrillation	8.88 × 10^−62^	2.0
Abnormal EKG	4.89 × 10^−44^	2.0
Ventricular tachycardia	6.28 × 10^−44^	2.6
Aortic dissection	9.09 × 10^−43^	2.2
Bicuspid aortic valve	1.60 × 10^−39^	2.2
Pointed chin	9.10 × 10^−35^	2.0
Aortic aneurysm	1.13 × 10^−34^	2.3
Prolonged QT interval	3.91 × 10^−34^	2.2

EKG, electrocardiogram; FDR, false discovery rate corrected. Additional highly enriched terms are included in Supplementary Table 3.
