# Supplementary material for: Genome-wide compendium and functional assessment of in vivo heart enhancers
Source: Nat Commun. 2016 Oct 5;7:12923. doi: 10.1038/ncomms12923 (PMC5059478; doi:10.1038/ncomms12923)
Supplement: Supplementary Information — Supplementary Figures 1-15, Supplementary Tables 1-11. Supplementary Notes 1-4 and Supplementary References [file ncomms12923-s1.pdf]

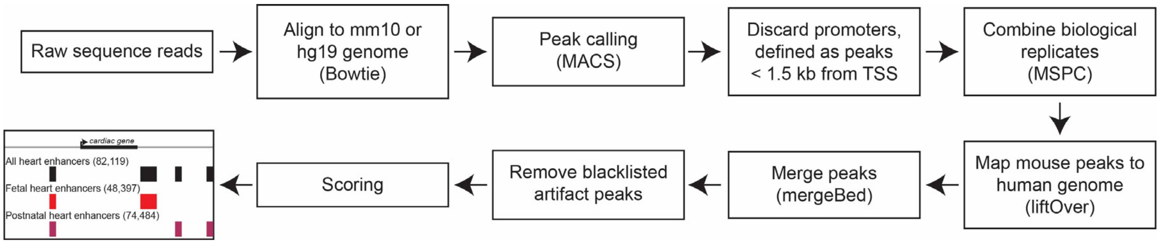

Supplementary Figure 1. Workflow for cardiac enhancer meta-analysis. Raw sequence reads for each ChIP-seq dataset were aligned to the mouse (mm10) or human (hg19) genome. After peak calling, promoter peaks (peaks <1.5 kb from a gene transcription start site) were discarded, and biological replicates were combined. Peaks from mouse data sets were lifted over to the human (hg19) genome, and peaks from all data sets were merged. We, and others, have previously observed that some H3K27ac peaks are highly enriched in nearly all datasets regardless of tissue type or developmental time point, and these are likely to be technical artifacts. We used an ENCODE-generated blacklist of such sites along with a similar list we generated to remove these artifacts prior to scoring (see **Methods**). This meta-analysis resulted in a total of 82,119 putative cardiac enhancers (48,397 in prenatal heart and 74,484 in postnatal heart).

a.

b.

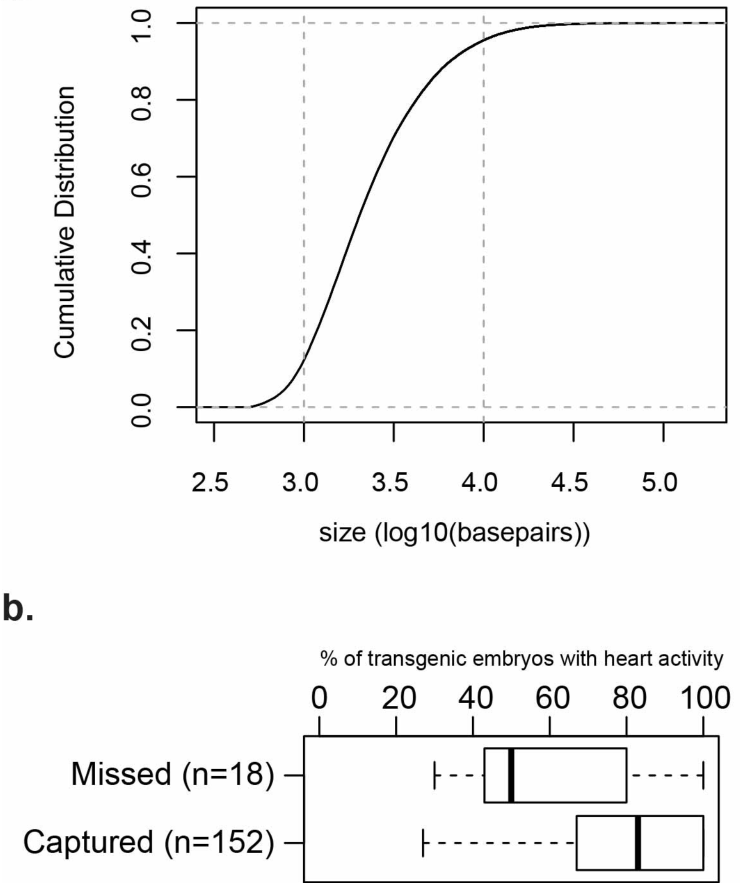

Supplementary Figure 2. Meta-analysis identification of putative heart enhancers. a) Size distribution of putative enhancers identified through meta-analysis. **b)** Transgenic enhancer assay reproducibility for VISTA heart enhancers captured or missed by the integrative analysis. Captured enhancers had higher percentages of transgenic embryos with reproducible heart staining (*P*-value = 9.5×10^{-4} by Mann-Whitney U Test). Panel **b** shows a standard boxplot with median, range, and quartile values plotted.

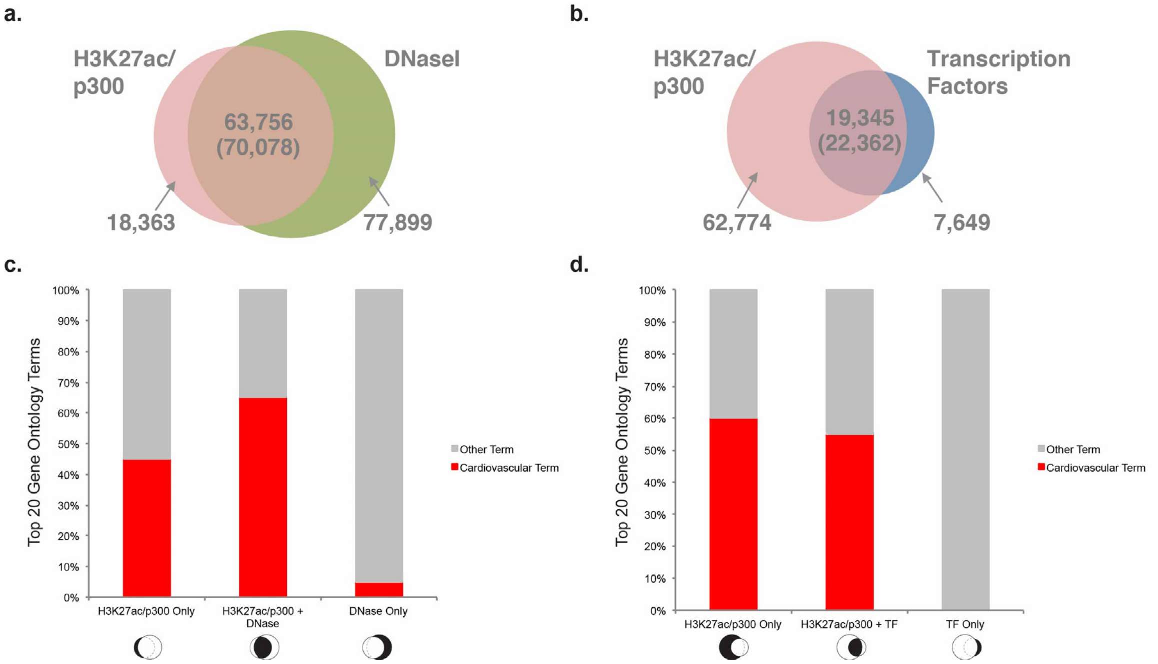

Supplementary Figure 3. DNase hypersensitivity (DHS) and transcription factor (TF) ChIP-seq do not identify substantially different loci from H3K27ac/p300. a,b) Similar integrative analyses using available mouse and human heart DHS **(a)** and cardiac TF data **(b)** identified many of the same loci as the integrative analysis using H3K27ac and p300. **c)** To assess whether the addition of DHS information is likely to substantially improve enhancer identification, we used GREAT to perform gene ontology (GO) analysis of those loci marked 1) only by H3K27ac/p300, 2) by both H3K27ac/p300 and DHS, and 3) only by DHS. There was clear enrichment of cardiovascular related GO terms for those loci marked by H3K27ac/p300, regardless of the DHS signature, while those sites with DHS alone showed no such association to cardiovascular biology. These results strongly suggest that the addition of existing DHS data would likely substantially increase the number of false positive elements while providing relatively little improvement to the accurate prediction of true heart enhancers. **d)** Same analysis as in **(c)** for TFs.

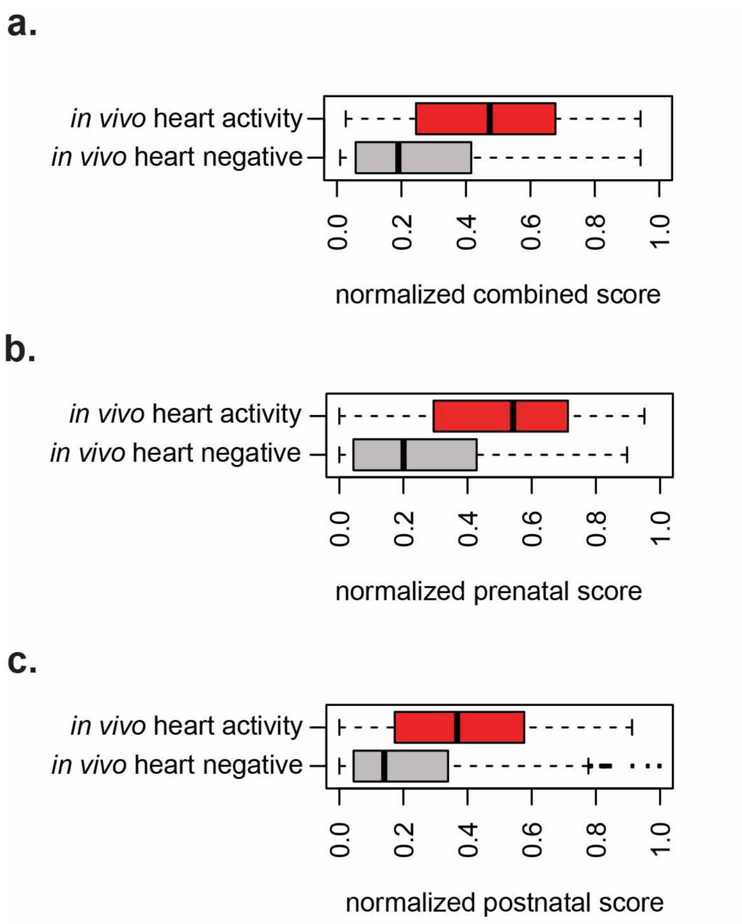

Supplementary Figure 4. Confidence scores correlate with *in vivo* validation. Putative enhancers identified by the heart meta-analysis that overlap VISTA heart enhancers have significantly higher combined **(a)**, prenatal **(b)**, and postnatal **(c)** confidence scores than the putative enhancers that overlap VISTA elements with no heart enhancer activity (*P*-values = 7.0×10^{-20}, 6.6×10^{-22}, and 3.7×10^{-14}, respectively, by Mann-Whitney U Test). All panels show standard boxplots with median, range, quartile, and outlier values plotted. See also **Supplementary Note 3**.

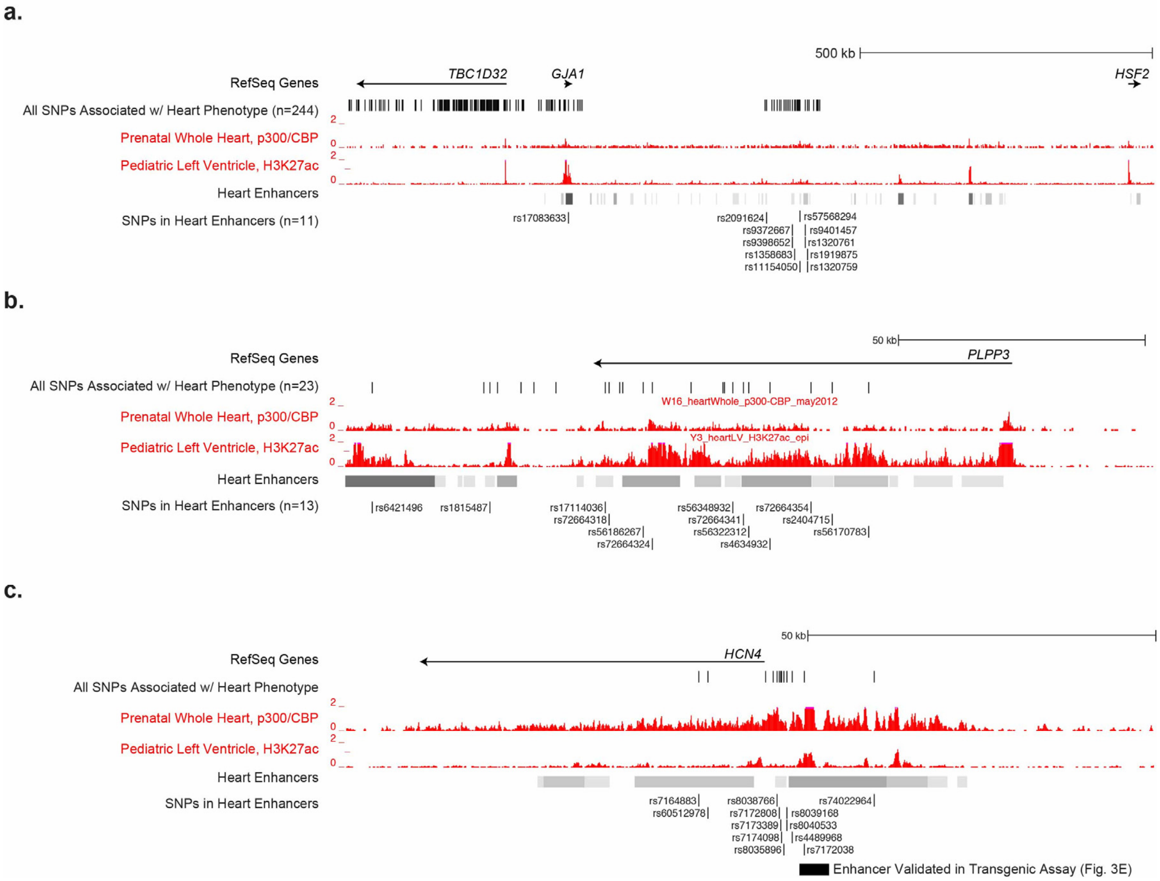

Supplementary Figure 5. Identifying GWAS variants in human heart enhancers. Shown are examples of three loci implicated in human heart phenotypes where associated SNPs fall within predicted cardiac enhancers, including: **a)** the 6q22 region near *GJA1* implicated in heart rate, **b)** the 1p32 region overlapping *PLPP3* implicated in coronary artery disease, and **c)** the 15q24 locus containing *HCN4* implicated in atrial fibrillation. For each locus, "All SNPs Associated w/ Heart Phenotype" includes all SNPs in the NHGRI-EBI GWAS Catalog that are associated with a heart phenotype AND all SNPs in strong LD ($r^2 \geq 0.8$) with the reported lead SNP(s). Heart enhancers are shown in grayscale indicating the overall confidence score of the enhancer (dark=higher, light=lower). At the bottom of each image ("SNPs in Heart Enhancers"), we indicate the names of those variants falling into putative heart enhancers. A candidate enhancer upstream of *HCN4* that overlaps with a human phenotype-associated variant was functionally validated and had strongly reproducible heart activity (**Fig. 3E**).

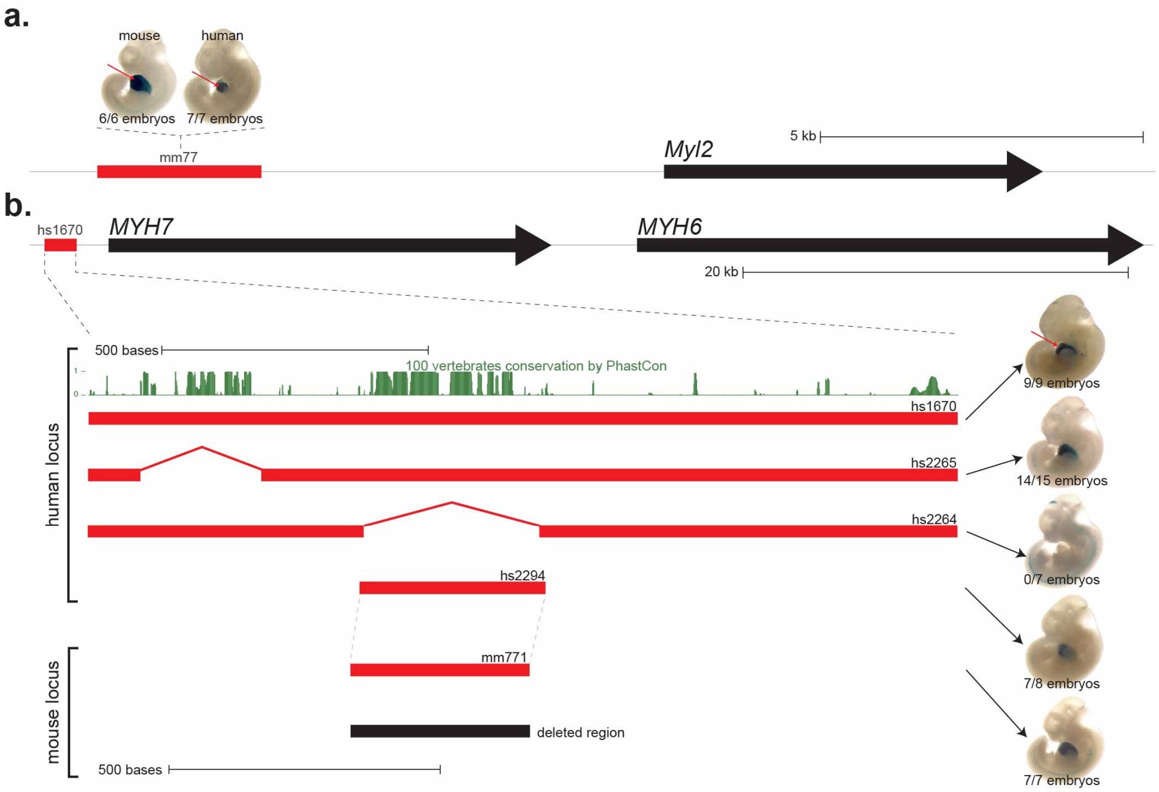

Supplementary Figure 6. Enhancers targeted for deletion are functionally conserved between human and mouse. a) Representative transgenic E11.5 mouse embryos carrying a *LacZ* reporter gene under the control of either the human or mouse homolog of mm77, a cardiac enhancer upstream of the *Myl2* gene. Both the human and mouse versions drive strong, highly reproducible transgene expression throughout the heart (red arrow). Embryo numbers indicate the number of embryos with reproducible heart expression of the transgene over the total number of transgenic embryos obtained. **b)** Representative transgenic E11.5 mouse embryos carrying a *LacZ* reporter gene under the control of either the human or mouse homolog of hs1670/mm771, a cardiac enhancer upstream of the *Myh7* gene. The full-length human homolog (hs1670) drives highly reproducible transgene expression throughout the heart at E11.5. To narrow down the minimum sequence necessary for heart enhancer activity, we generated allelic variants of hs1670 that were missing one of two highly conserved sequences (hs2265, hs2264). Allele hs2265 maintained strong heart enhancer activity, in contrast to allele hs2264, indicating that the sequence necessary for heart enhancer activity resides in the ~350 basepairs removed from hs2264. Both the human (hs2294) and mouse (mm771) homologs of this ~350 basepair sequence were sufficient for highly reproducible enhancer activity in the heart, so only this minimal region was targeted for deletion (black bar).

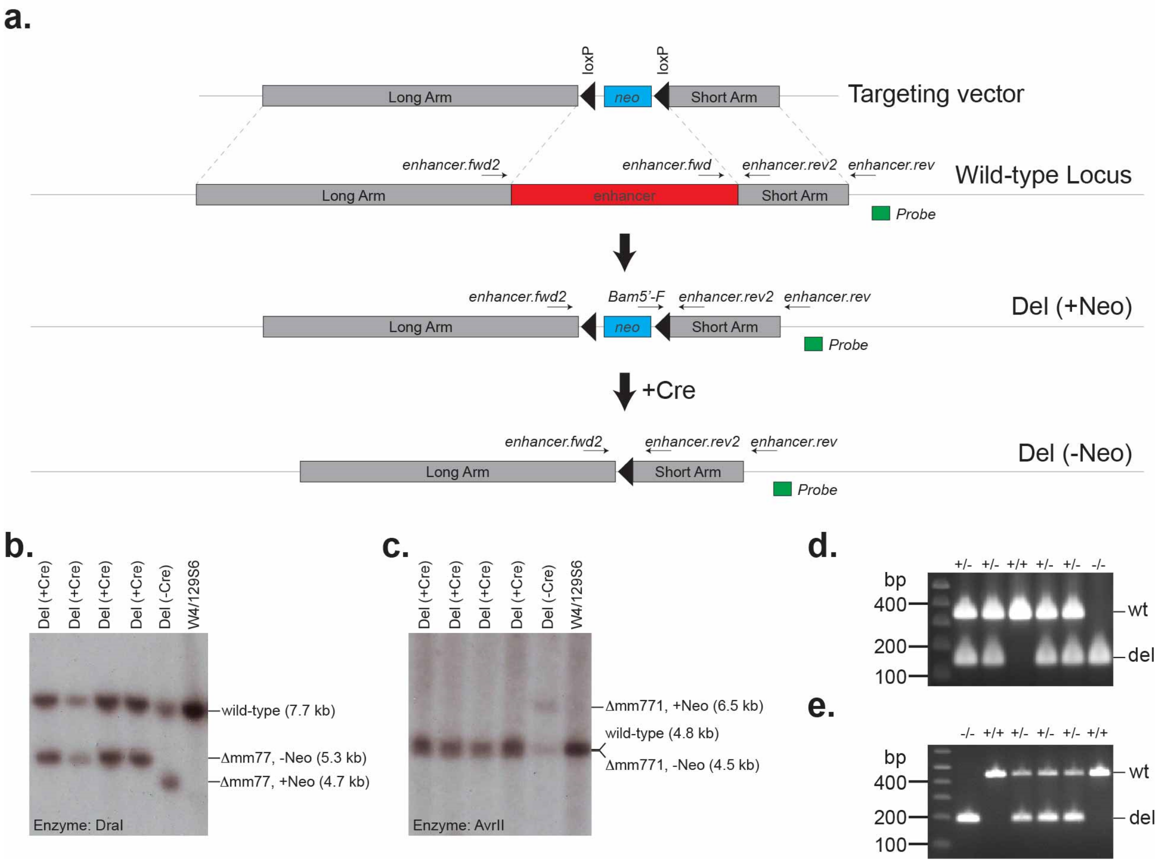

Supplementary Figure 7. Generation and validation of enhancer knockout mice. a) Targeting strategy to generate enhancer-null mice. The targeting vector, containing a Neomycin resistance cassette (*neo*) flanked by loxP sites (black arrow heads) and homology arms, was introduced into wild-type mouse embryonic stem cells, where it integrated into the genome, replacing the targeted enhancer with the *neo* cassette. Introduction of a plasmid encoding a Cre-recombinase into correctly targeted cells was used to remove the *neo* gene, leaving a single loxP site in place of the enhancer. Primers used for genotyping and for validating the integration and *neo*-excision events are shown as thin black arrows. The site of the Southern probe used to validate correct targeting is shown as a green box. Schematic locus is not drawn to scale. **b,c)** Southern blot validation of mm77 **(b)** and mm771 **(c)** targeted embryonic stem cells. "Enzyme" indicates the restriction enzyme used to digest the DNA. Unmodified embryonic stem cells are shown in the lanes marked W4/129S6. kb: kilobases. **d,e)** Representative PCR-based genotyping for mice wild-type (+/+), heterozygous (+/-), or homozygous null (-/-) for the mm77 **(d)** or mm771 **(e)** enhancer. bp: base pairs.

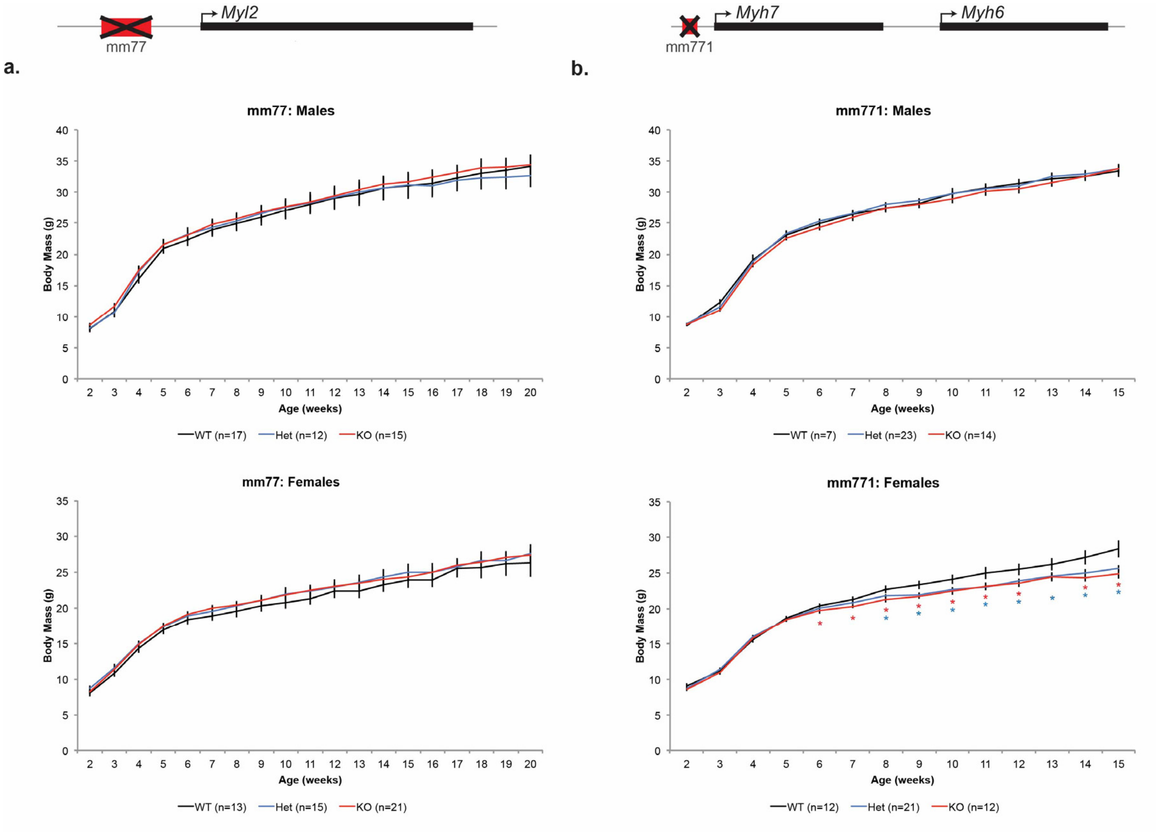

Supplementary Figure 8. Growth curves for enhancer deletion mice. Average body mass for male and female mice homozygous wild-type (WT), heterozygous (Het), or homozygous null (KO) for enhancer mm77 **(a)** or mm771 **(b)**. Error bars indicate SEM. "*" indicates significantly reduced body mass of homozygous null (red) or heterozygous (blue) mice compared to wild-type (P<0.05, one-tailed t-test).

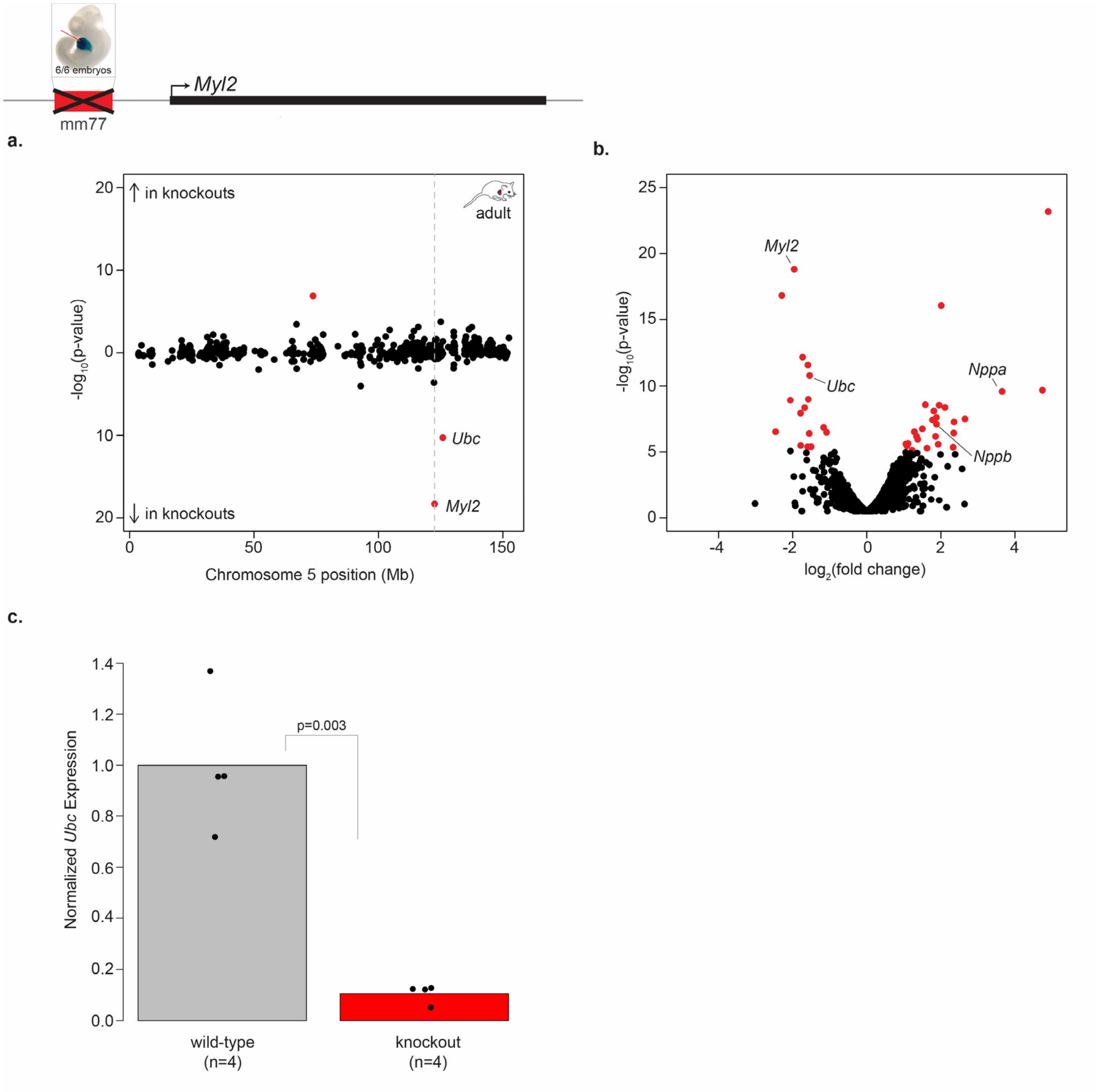

Supplementary Figure 9. Reduced *Myl2* expression persists into adulthood. Gene expression changes for chromosome 5 genes **(a)** and genome-wide **(b)** for adult mice homozygous null (n=4) compared to homozygous wild-type (n=4) for the mm77 enhancer. In adulthood, homozygous null mice show upregulation of *Nppa* and *Nppb*, which are biomarkers of heart failure. Red points **(a,b)** indicate statistically significant up- or downregulation ($P < 0.01$ using an FDR < 5%, see **Methods** for details). Dashed gray line (**a**) indicates the position of the enhancer. **c)** qPCR validation for *Ubc* gene expression, which was significantly decreased in knockout animals. Bars indicate gene expression means, points indicate individual animals, values were normalized to actin and then to the wild-type mean, and *P*-value was calculated using a one-tailed *t*-test. RNA-seq **(a,b)** and qPCR validation **(c)** was performed on ventricular heart tissue dissected from mice approximately six months of age.

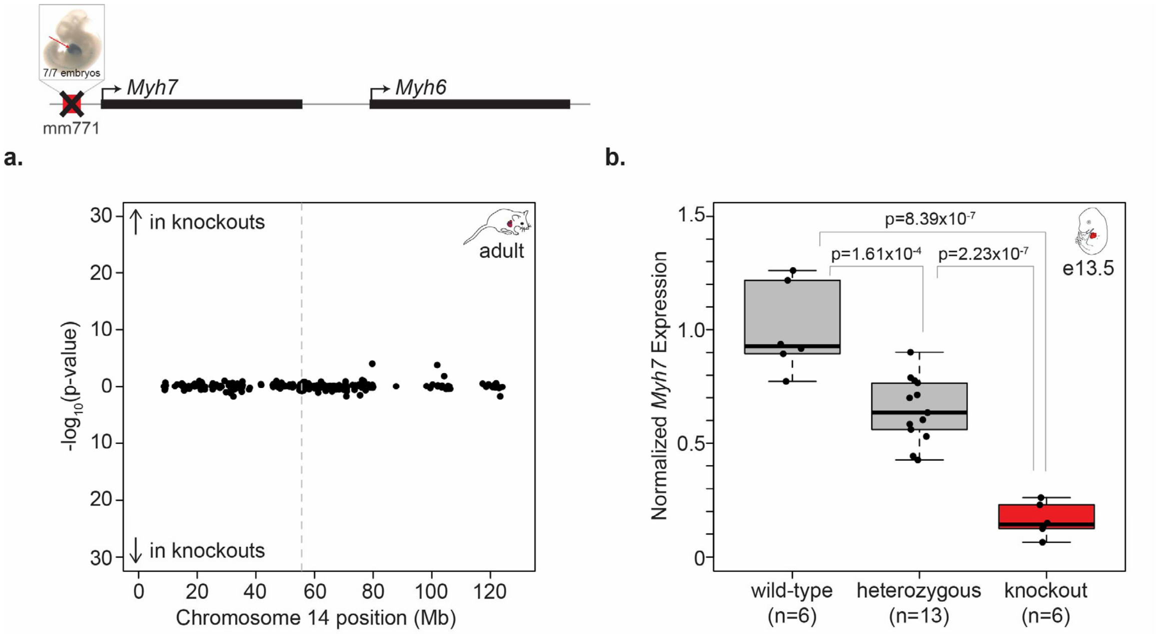

Supplementary Figure 10. Loss of mm771 results in decreased *Myh7* expression in embryogenesis but not adulthood. a) Significance of gene expression changes in adult heart for all genes on chromosome 14. RNA-seq was performed on the ventricular portion of the heart for two homozygous null and two wild-type animals. Points indicate individual genes. -log10(p-values) above and below 0 indicate genes upregulated or downregulated, respectively, in knockout relative to wild-type animals. The dashed gray line indicates the position of the mm771 enhancer. Mb: megabases. **b)** Normalized *Myh7* mRNA levels measured by quantitative RT-PCR in whole hearts collected from E13.5 mouse embryos wild-type, heterozygous or homozygous null (knockout) for the mm771 enhancer. Boxplots indicate the median, range, and quartile values for each data set, and points indicate individual biological replicates. *Myh7* mRNA levels were normalized to actin and then normalized to the mean of the wild-type class, which was set at 1. For **b**, *P*-values calculated by one-tailed *t*-test.

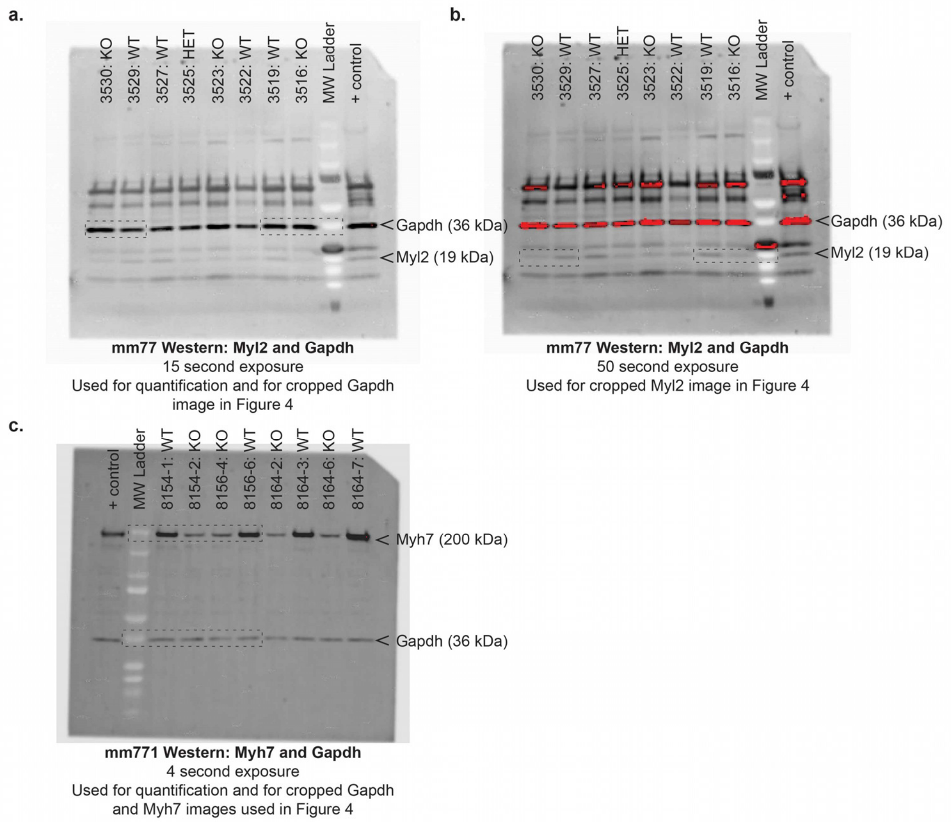

Supplementary Figure 11: Uncropped versions of western blots shown in Figure 4. a) and **b)** show the same blot imaged with different exposure times. Black dashed boxes indicate the bands that are shown in **Fig. 4**. Bands for 3529 (WT) and 3530 (KO) were vertically mirrored in **Fig. 4** to facilitate comparison across replicates. Red pixels indicate signal saturation. For all blots, MW Ladder is Bio-Rad Precision Plus Protein Standards. KO: homozygous null, WT: wild-type, HET: heterozygous.

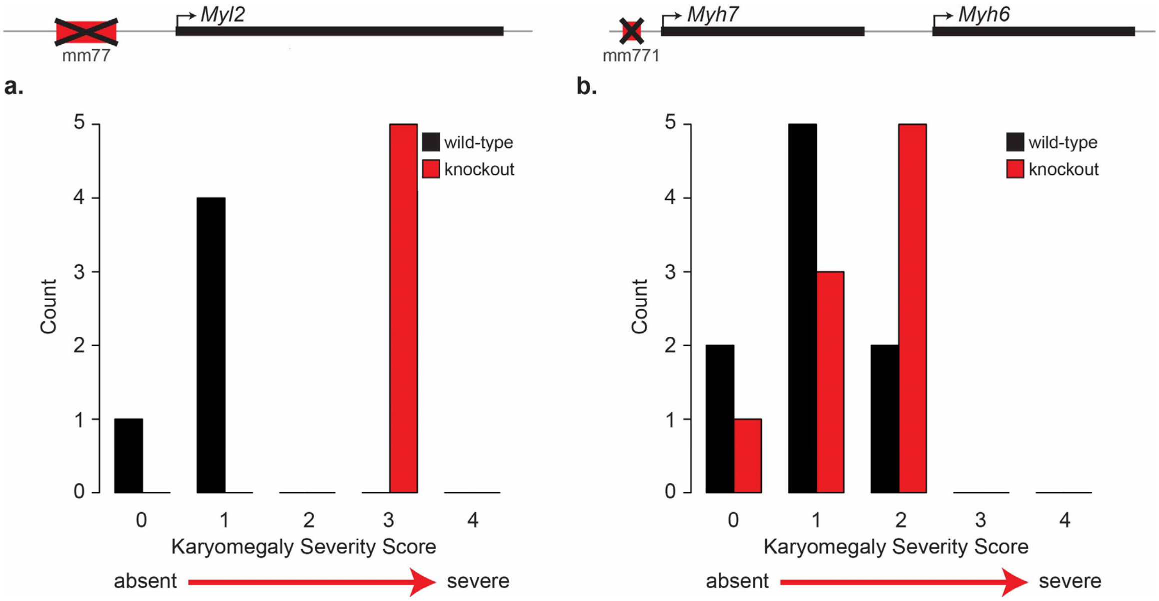

Supplementary Figure 12. Severity of cardiac karyomegaly in enhancer null mice. Severity of myocardiocyte karyomegaly observed in the hearts of mice wild-type or homozygous null for the mm77 **(a)** or mm771 **(b)** enhancer. Cardiac tissue was scored by a genotype-blind pathologist from 0 (absent) to 4 (severe). Mice homozygous null for mm77 showed elevated myocardiocyte karyomegaly compared to wild-type littermates (P = 0.024 by paired one-tailed Wilcoxon rank-sum test). Differences in karyomegaly severity observed between mm771 null and wild-type littermates were not statistically significant (P = 0.101).

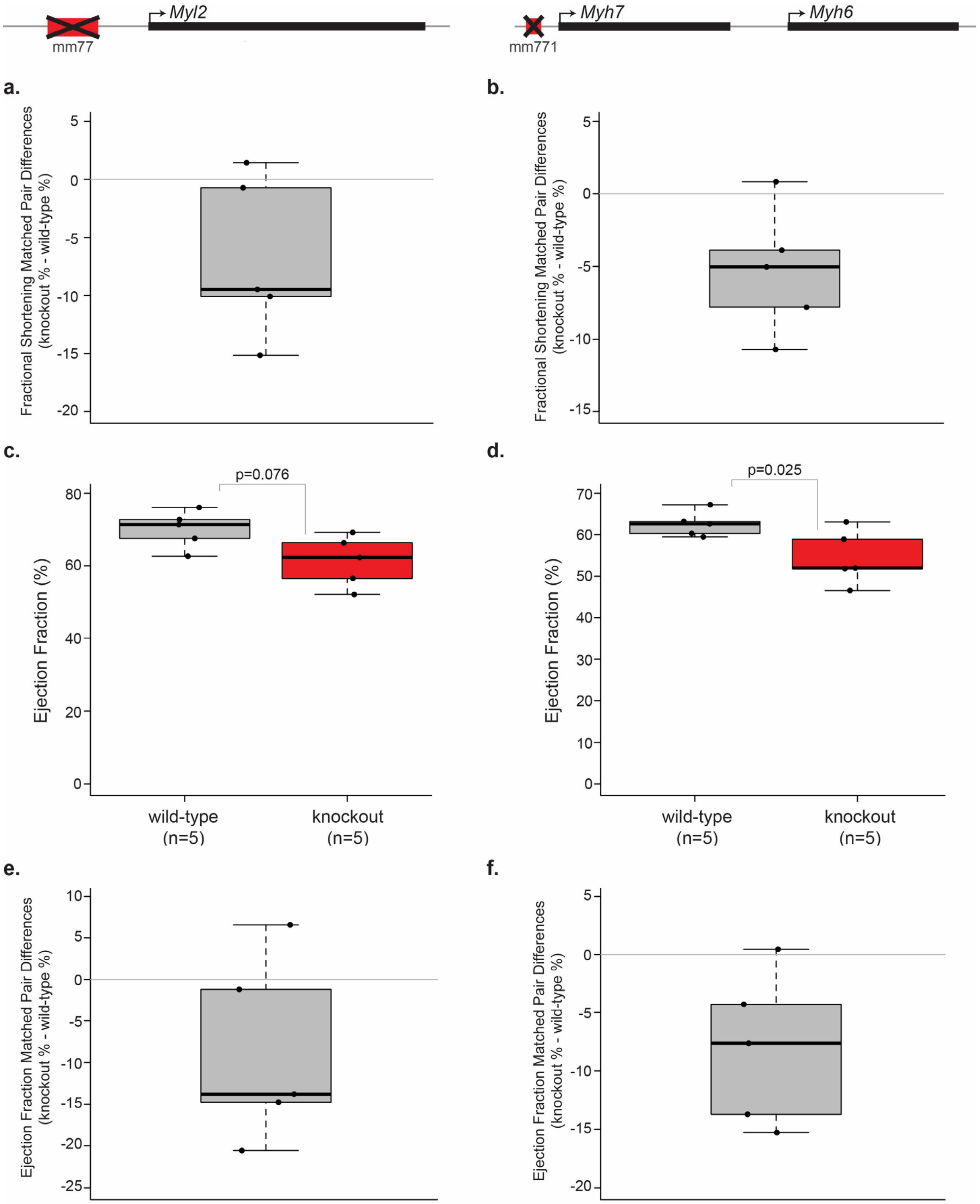

Supplementary Figure 13. Loss of cardiac enhancers results in decreased heart function. a,b) Pairwise differences in fractional shortening between matched homozygous null and wild-type littermate pairs for the mm77 **(a)** and mm771 **(b)** enhancer deletion lines. Raw fractional shortening values for all mice are shown in **Figures 4a** and **4b**. **c,d)** Ejection fraction measured by echocardiography for mice wild-type or homozygous null (knockout) for the mm77 **(c)** or mm771 **(d)** enhancer. Mice homozygous null for mm77 show a statistically not significant tendency toward decreased ejection fraction, whereas mice homozygous null for mm771 show a significant decrease. *P*-values calculated by paired one-tailed *t*-test. **e,f)** Pairwise differences in ejection fraction between matched littermate pairs for mm77 **(e)** and mm771 **(f)**. All panels show standard boxplots with median, range, individual samples (points), and quartile values plotted.

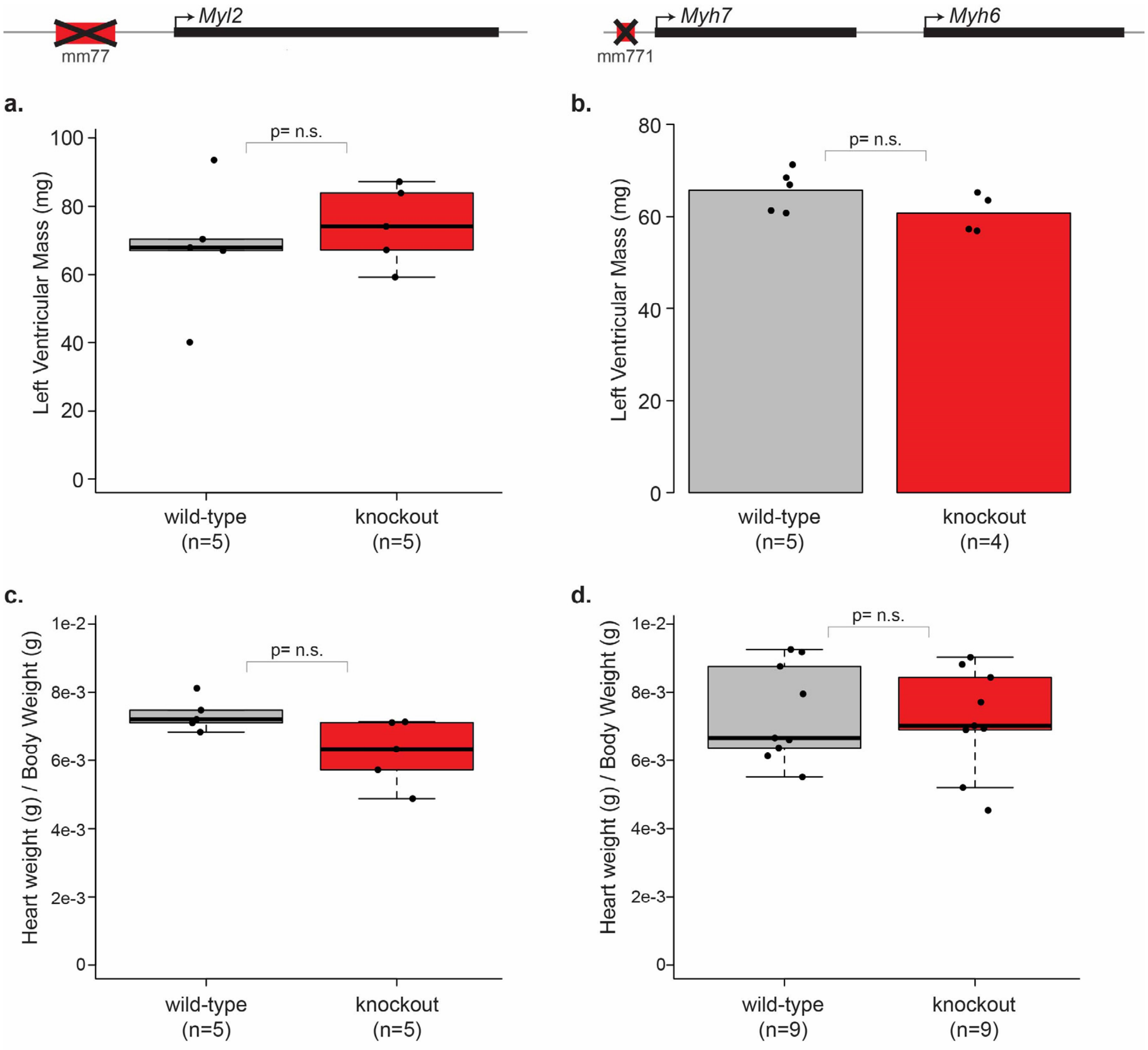

Supplementary Figure 14. Loss of heart enhancers does not cause obvious hypertrophy. **a,b)** Left ventricular mass for postnatal mice homozygous wild-type or homozygous null (knockout) for the mm77 **(a)** or mm771 **(b)** enhancer. Left ventricular mass was not significantly greater for enhancer null mice relative to their wild-type littermates (one-tailed paired *t*-test). **c,d)** Heart weight, normalized to body weight, for postnatal mice homozygous wild-type or homozygous null for the mm77 **(c)** or mm771 **(d)** enhancer. Normalized heart weight was not significantly greater for enhancer null mice relative to their wild-type littermates (one-tailed paired *t*-test). n.s.: not significant. Boxplots (**a,c,d**) show median, range, and quartile values. Bar graphs (**b**) show sample mean. In all panels, points indicate individual samples.

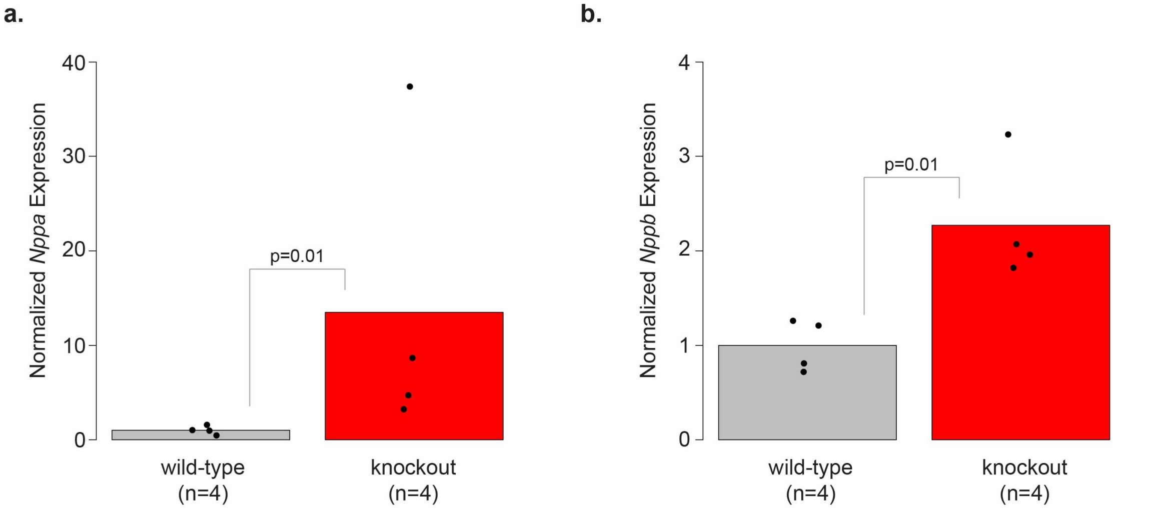

Supplementary Figure 15. qPCR validation of *Nppa* and *Nppb* upregulation in mm77-null mice. Normalized *Nppa* (a) and *Nppb* (b) mRNA levels measured by quantitative RT-PCR in ventricular heart tissue of adult mice homozygous wild-type or homozygous null for mm77. Bars indicate the mean for each data set, and points indicate individual biological replicates. *Nppa* and *Nppb* mRNA levels were normalized to actin and then normalized to the mean of the wild-type class, which was set at 1. *P*-values calculated by one-tailed Wilcoxon rank-sum test.

Supplementary Tables

Supplementary Table 1. Human and mouse heart H3K27ac and p300 ChIP-seq datasets used in this study

ID	Organism	Fetal / Postnatal	Stage / Age	Whole heart / Region	ChIP	Notes	Ref	Source
1	Human	Fetal	Gestational age: 16 weeks	Whole	p300/CBP	no input sample	1	http://www.ncbi.nlm.nih.gov/geo/query/acc.cgi?acc=GSE32587
2	Human	Postnatal	3 years	Left Ventricle	H3K27ac	-	2	http://www.ncbi.nlm.nih.gov/geo/roadmap/epigenomics/
3	Human	Postnatal	3 years	Right Ventricle	H3K27ac	-	2	http://www.ncbi.nlm.nih.gov/geo/roadmap/epigenomics/
4	Human	Postnatal	30 years	Aorta	H3K27ac	-	2	http://www.ncbi.nlm.nih.gov/geo/roadmap/epigenomics/
5	Human	Postnatal	34 years	Aorta	H3K27ac	-	2	http://www.ncbi.nlm.nih.gov/geo/roadmap/epigenomics/
6	Human	Postnatal	34 years	Left Ventricle	H3K27ac	-	2	http://www.ncbi.nlm.nih.gov/geo/roadmap/epigenomics/
7	Human	Postnatal	34 years	Right Atrium	H3K27ac	-	2	http://www.ncbi.nlm.nih.gov/geo/roadmap/epigenomics/
8	Human	Postnatal	34 years	Right Ventricle	H3K27ac	-	2	http://www.ncbi.nlm.nih.gov/geo/roadmap/epigenomics/
9	Human	Postnatal	45 years	Septum	p300/CBP	Adult ischemic heart, no input sample	1	http://www.ncbi.nlm.nih.gov/geo/query/acc.cgi?acc=GSE32587
10	Mouse	Fetal	E11.5	Whole	H3K27ac	2 biological replicates	3	http://www.ncbi.nlm.nih.gov/geo/query/acc.cgi?acc=GSE52386
11	Mouse	Fetal	E11.5	Whole	H3K27ac	2 biological replicates	n/a	https://www.encodeproject.org/experiments/ENCSR222IHX/
12	Mouse	Fetal	E11.5	Whole	p300	-	4	http://www.ncbi.nlm.nih.gov/geo/query/acc.cgi?acc=GSM559652
13	Mouse	Fetal	E13.5	Whole	H3K27ac	2 biological replicates	n/a	https://www.encodeproject.org/experiments/ENCSR699XHY/
14	Mouse	Fetal	E14.5	Whole	H3K27ac	-	3	http://www.ncbi.nlm.nih.gov/geo/query/acc.cgi?acc=GSE52386
15	Mouse	Fetal	E14.5	Whole	H3K27ac	2 biological replicates	5	http://www.ncbi.nlm.nih.gov/geo/query/acc.cgi?acc=GSM1000137
16	Mouse	Fetal	E14.5	Whole	H3K27ac	-	6	http://www.ncbi.nlm.nih.gov/geo/query/acc.cgi?acc=GSM851290
17	Mouse	Fetal	E15.5	Whole	H3K27ac	2 biological replicates	n/a	https://www.encodeproject.org/experiments/ENCSR574VME/

ID	Organism	Fetal / Postnatal	Stage / Age	Whole heart / Region	ChIP	Notes	Ref	Source
18	Mouse	Fetal	E16.5	Whole	H3K27ac	2 biological replicates	n/a	https://www.encodeproject.org/experiments/ENCSR846PJO/
19	Mouse	Fetal	E17.5	Whole	H3K27ac	-	3	http://www.ncbi.nlm.nih.gov/geo/query/acc.cgi?acc=GSE52386
20	Mouse	Postnatal	P0	Whole	H3K27ac	-	3	http://www.ncbi.nlm.nih.gov/geo/query/acc.cgi?acc=GSE52386
21	Mouse	Postnatal	P0	Whole	H3K27ac	2 biological replicates	n/a	https://www.encodeproject.org/experiments/ENCSR675HDX/
22	Mouse	Postnatal	P5	Whole	p300	-	7	http://www.ncbi.nlm.nih.gov/geo/query/acc.cgi?acc=GSM862699
23	Mouse	Postnatal	P7	Whole	H3K27ac	-	3	http://www.ncbi.nlm.nih.gov/geo/query/acc.cgi?acc=GSE52386
24	Mouse	Postnatal	P21	Whole	H3K27ac	-	3	http://www.ncbi.nlm.nih.gov/geo/query/acc.cgi?acc=GSE52386
25	Mouse	Postnatal	P56	Whole	p300	2 biological replicates	6	http://www.ncbi.nlm.nih.gov/geo/query/acc.cgi?acc=GSM722695
26	Mouse	Postnatal	P56	Whole	H3K27ac	2 biological replicates	5	http://www.ncbi.nlm.nih.gov/geo/query/acc.cgi?acc=GSM1000093
27	Mouse	Postnatal	P56	Whole	H3K27ac	-	3	http://www.ncbi.nlm.nih.gov/geo/query/acc.cgi?acc=GSE52386
28	Mouse	Postnatal	P56	Whole	H3K27ac	-	6	http://www.ncbi.nlm.nih.gov/geo/query/acc.cgi?acc=GSM851273

All datasets used are publicly available (Source), and most have been previously published (Ref). Mouse time points are given as embryonic (e.g. E11.5) or postnatal (e.g. P56) day. Biological replicates from the same paper, as indicated in "Notes", were included and ultimately integrated into a single dataset.

Supplementary Table 2: Percentage of mouse heart enhancer peaks that are conserved to human

Mark	Stage	# Mouse Elements (mm9)	# Mouse Elements That Lift to Human	% Mouse Elements That Lift To Human	# Mouse Elements Overlapping Human Element	% of Mouse Elements Overlapping Human Element
H3K27ac	E11.5	24827	20442	82.34%	11474	46.22%
p300	E11.5	2031	1642	80.85%	895	44.07%
H3K27ac	E13.5	28944	23733	82.00%	13539	46.78%
H3K27ac	E14.5	28965	23861	82.38%	13608	46.98%
H3K27ac	E15.5	26278	21590	82.16%	12953	49.29%
H3K27ac	E16.5	20293	16916	83.36%	10760	53.02%
H3K27ac	E17.5	20191	16828	83.34%	10442	51.72%
H3K27ac	P0	40557	32669	80.55%	17837	43.98%
p300	P5	1868	1585	84.85%	1115	59.69%
H3K27ac	P7	45473	35963	79.09%	19051	41.90%
H3K27ac	P21	12076	9910	82.06%	5911	48.95%
H3K27ac	P56	37075	29940	80.76%	17312	46.69%
p300	P56	32259	25542	79.18%	13573	42.08%
**Average**				**81.76%**		**47.80%**

Approximately 80% of H3K27ac or p300 peaks identified in mouse heart tissue could be lifted over to the human (hg19) genome using liftOver with a minMatch of 0.1 in both directions (mouse to human and human to mouse). Nearly 50% of all putative enhancer intervals identified in mouse heart overlapped with an enhancer interval identified in human heart (i.e. are functionally conserved between mouse and human).

Supplementary Table 3: Most highly enriched ontology terms for integrative analysis catalog of human heart enhancers

**Human Phenotype**			
**Term Name**	**Binomial Raw P-value**	**Binomial FDR Q-Value**	**Binomial Fold Enrichment**
Cardiac arrest	1.66E-91	6.81E-89	2.026
Sudden cardiac death	2.04E-91	7.84E-89	2.029
Sudden death	1.13E-86	3.67E-84	3.115
Syncope	6.77E-67	1.22E-64	2.240
Atrial fibrillation	5.64E-64	8.88E-62	2.009
Abnormal EKG	6.12E-46	4.89E-44	2.002
Ventricular tachycardia	7.97E-46	6.28E-44	2.610
Aortic dissection	1.26E-44	9.09E-43	2.203
Bicuspid aortic valve	2.34E-41	1.60E-39	2.188
Pointed chin	1.60E-36	9.10E-35	2.027
Aortic aneurysm	2.00E-36	1.13E-34	2.306
Prolonged QT interval	7.31E-36	3.91E-34	2.185
Polyneuropathy	1.67E-34	8.19E-33	2.152
Generalized muscle weakness	2.21E-34	1.05E-32	2.207
Spinal rigidity	4.16E-34	1.94E-32	2.493

Mouse Phenotype			
Term Name	Binomial Raw P-value	Binomial FDR Q-Value	Binomial Fold Enrichment
Abnormal cell adhesion	2.10E-138	1.15E-136	2.223
Abnormal sarcomere morphology	3.06E-104	1.13E-102	2.128
Abnormal fourth branchial arch artery morphology	1.21E-68	2.73E-67	2.078
Disorganized yolk sac vascular plexus	5.35E-54	9.38E-53	2.096
Abnormal fetal cardiomyocyte morphology	1.04E-50	1.72E-49	2.069
Decreased vascular endothelial cell number	7.64E-46	1.11E-44	2.631
Abnormal brain meninges morphology	1.06E-42	1.42E-41	2.249
Enhanced wound healing	2.97E-40	3.78E-39	2.134
Abnormal terminal bronchiole morphology	4.56E-40	5.78E-39	2.109
Ruffled hair	5.61E-39	6.92E-38	2.118
Asymmetric snout	5.25E-38	6.29E-37	2.879
Decreased placenta weight	1.33E-37	1.57E-36	2.096
Abnormal white adipose tissue physiology	2.25E-37	2.63E-36	2.321
Abnormal branching involved in terminal bronchiole morphogenesis	3.89E-37	4.53E-36	2.141
Abnormal macrophage apoptosis	1.09E-34	1.16E-33	2.307

GO Biological Process			
Term Name	Binomial Raw P-value	Binomial FDR Q-Value	Binomial Fold Enrichment
Actomyosin structure organization	1.98E-117	7.70E-116	2.141
Myofibril assembly	1.30E-104	4.27E-103	2.222
Negative regulation of transforming growth factor beta receptor signaling pathway	3.50E-100	1.11E-98	2.016
Sarcomere organization	9.33E-98	2.91E-96	2.806
Cell-substrate junction assembly	2.24E-74	5.51E-73	2.255
Establishment of protein localization to plasma membrane	1.16E-69	2.71E-68	2.166
Extrinsic apoptotic signaling pathway	7.17E-63	1.55E-61	2.017
Regulation of nuclear-transcribed mRNA catabolic process, deadenylation-dependent decay	1.11E-55	2.14E-54	2.783
Positive regulation of nuclear-transcribed mRNA catabolic process, deadenylation-dependent decay	3.85E-55	7.31E-54	2.803
Cardiac muscle hypertrophy	1.18E-54	2.22E-53	2.118
Regulation of ventricular cardiac muscle cell action potential	2.99E-54	5.59E-53	2.323
Positive regulation of mRNA catabolic process	2.43E-53	4.45E-52	2.623
Regulation of membrane depolarization	2.22E-50	3.85E-49	2.175
Positive regulation of protein dephosphorylation	2.46E-50	4.26E-49	2.767
Muscle hypertrophy	3.31E-50	5.71E-49	2.018

Analysis was performed using the GREAT program. Only the top 15 most enriched terms for each classification performed by GREAT are listed.

Supplementary Table 4: Human and mouse heart transcription factor ChIP-seq and DNase hypersensitivity datasets used in this study

ID	Organism	Fetal / Postnatal	Stage / Age	ChIP/Dnase	Notes	Ref	Source
1	Human	Fetal	96 days	DNase HS	male	2	http://www.ncbi.nlm.nih.gov/geo/query/acc.cgi?acc=GSM530654
2	Human	Fetal	101 days	DNase HS	-	2	http://www.ncbi.nlm.nih.gov/geo/query/acc.cgi?acc=GSM530661
3	Human	Fetal	117 days	DNase HS	female	2	http://www.ncbi.nlm.nih.gov/geo/query/acc.cgi?acc=GSM665809
4	Human	Fetal	96 days	DNase HS	male	2	http://www.ncbi.nlm.nih.gov/geo/query/acc.cgi?acc=GSM665811
5	Human	Fetal	103 days	DNase HS	female	2	http://www.ncbi.nlm.nih.gov/geo/query/acc.cgi?acc=GSM665814
6	Human	Fetal	103 days	DNase HS	male	2	http://www.ncbi.nlm.nih.gov/geo/query/acc.cgi?acc=GSM665817
7	Human	Fetal	147 days	DNase HS	female	2	http://www.ncbi.nlm.nih.gov/geo/query/acc.cgi?acc=GSM665824
8	Human	Fetal	110 days	DNase HS	female	2	http://www.ncbi.nlm.nih.gov/geo/query/acc.cgi?acc=GSM665830
9	Human	Fetal	110 days	DNase HS	female	2	http://www.ncbi.nlm.nih.gov/geo/query/acc.cgi?acc=GSM665831
10	Human	Fetal	105 days	DNase HS	female	2	http://www.ncbi.nlm.nih.gov/geo/query/acc.cgi?acc=GSM774203
11	Human	Fetal	91 days	DNase HS	female	2	http://www.ncbi.nlm.nih.gov/geo/query/acc.cgi?acc=GSM817220
12	Human	Fetal	120 days	DNase HS	male	2	http://www.ncbi.nlm.nih.gov/geo/query/acc.cgi?acc=GSM878630
13	Human	Fetal	76 days	DNase HS	male	n/a	https://www.encodeproject.org/experiments/ENCSR705CNJ/
14	Human	Fetal	72 days	DNase HS	male	n/a	https://www.encodeproject.org/experiments/ENCSR705CNJ/
15	Human	Postnatal	3 years	DNase HS	male	2	http://www.ncbi.nlm.nih.gov/geo/query/acc.cgi?acc=GSM1027322
16	Mouse	Postnatal	P56	DNase HS	-	5	https://www.encodeproject.org/experiments/ENCSR000CNE/
17	Mouse	Fetal	E12.5	Gata4	endogenous	8	http://www.ncbi.nlm.nih.gov/geo/query/acc.cgi?acc=GSE52123
18	Mouse	Fetal	E12.5	Gata4	FLAG-tagged	8	http://www.ncbi.nlm.nih.gov/geo/query/acc.cgi?acc=GSE52123

ID	Organism	Fetal / Postnatal	Stage / Age	ChIP/Dnase	Notes	Ref	Source
19	Mouse	Fetal	E11.5	Smarca4 (Brg1)	FLAG-tagged	9	http://www.ncbi.nlm.nih.gov/geo/query/acc.cgi?acc=GSE37151
20	Mouse	Postnatal	P56	Tbx20	GFP-tagged	10	http://www.ncbi.nlm.nih.gov/geo/query/acc.cgi?acc=GSE30943
21	Mouse	Postnatal	Adult	Brd4	-	11	http://www.ncbi.nlm.nih.gov/geo/query/acc.cgi?acc=GSE46668
22	Mouse	Postnatal	6-10 weeks	Gata4	endogenous	8	http://www.ncbi.nlm.nih.gov/geo/query/acc.cgi?acc=GSE52123
23	Mouse	Postnatal	6-10 weeks	Gata4	FLAG-tagged	8	http://www.ncbi.nlm.nih.gov/geo/query/acc.cgi?acc=GSE52123
24	Mouse	Postnatal	Adult	Gata4	-	7	http://www.ncbi.nlm.nih.gov/geo/query/acc.cgi?acc=GSE35151
25	Mouse	Postnatal	Adult	Tbx3	-	7	http://www.ncbi.nlm.nih.gov/geo/query/acc.cgi?acc=GSE35151
26	Mouse	Postnatal	Adult	Nkx2-5	-	7	http://www.ncbi.nlm.nih.gov/geo/query/acc.cgi?acc=GSE35151

All datasets used are publicly available (Source) and most have been previously published (Ref). Mouse time points are given as embryonic (e.g. E12.5) or postnatal (e.g. P56) day. PMID refers to the PubMed identifier for the reference paper.

Supplementary Table 5: Comparison of this integrative analysis to other enhancer prediction methods

Method	Enhancer set	AUC	Equivalent AUC for unsupervised H3K27ac/p300	Notes
EMERGE Supervised	VISTA Heart+ (mouse) vs VISTA negative	0.780	0.747	Analysis reported in paper
EMERGE Supervised	VISTA Heart+ (mouse) vs VISTA+ in other tissues	0.910	0.872	Analysis reported in paper
EMERGE Supervised	VISTA Heart+ (human) vs VISTA negative	0.860	0.854	Analysis reported in paper
EMERGE Supervised	VISTA Heart+ (human) vs VISTA+ in other tissues	0.860	0.871	Analysis reported in paper
EMERGE Supervised*	VISTA Heart+ (mouse and human) vs the rest of VISTA	0.860	0.865	New EMERGE analysis using identical set of H3K27ac and p300 data
EMERGE Unsupervised*	VISTA Heart+ (mouse and human) vs the rest of VISTA	0.864	0.865	New EMERGE analysis using identical set of H3K27ac and p300 data
EnhancerFinder	VISTA Heart+ (mouse and human) vs the rest of VISTA	0.850	0.865	Analysis reported in paper

Performance for our unsupervised integrative analysis was overall very similar to that of previous supervised methods, as indicated by similar area under the curve (AUC) for receiver operating characteristic curves. Unless noted with a "*", AUCs for EMERGE and EnhancerFinder are those reported for the heart enhancer predictions performed in their corresponding papers[12,13]. Those entries marked with a "*" indicate where we performed a new EMERGE analysis using the same H3K27ac and p300 datasets included in our unsupervised integrative analysis.

Supplementary Table 6: Summary of *in vivo* enhancers near heart disease genes

VISTA ID	Coordinates (hg19)<br>Nearest HD gene	Whole Embryo	Heart	Heart Histology	Annotation<br>(Reproducibility)
hs2125	chr1:11,945,528-11,949,490<br>*NPPB*	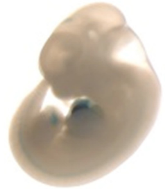	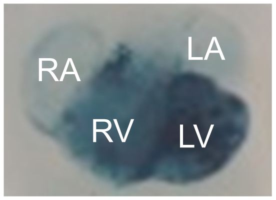	n/a	Heart (6/7)
hs2126	chr1:147,220,710-147,225,901<br>*GJA5*	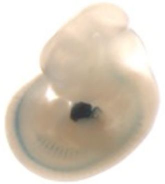	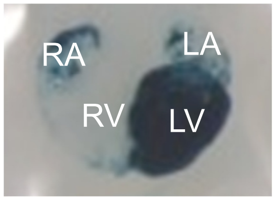	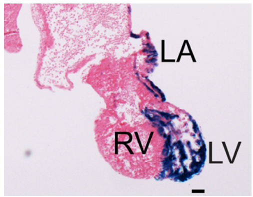	Heart (5/6)
hs2129	chr1:156,072,303-156,076,849<br>*LMNA*	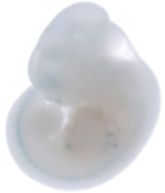	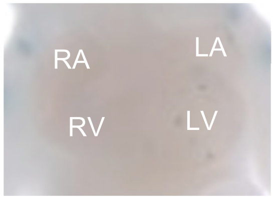	n/a	Heart (14/17)
hs2133	chr1:230,886,439-230,890,074<br>*AGT*	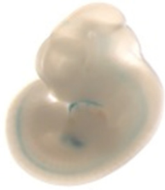	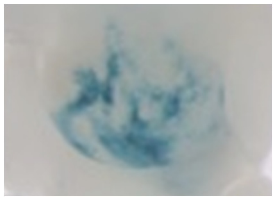	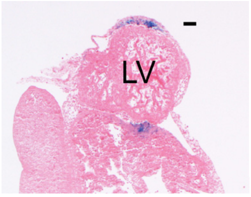	Heart (7/9),<br>Unidentified abdominal structure (6/9)

VISTA ID	Coordinates (hg19)<br>Nearest HD gene	Whole Embryo	Heart	Heart Histology	Annotation<br>(Reproducibility)
hs2135	chr1:236,851,383-<br>236,853,715<br><br>*ACTN2*	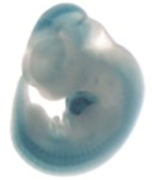	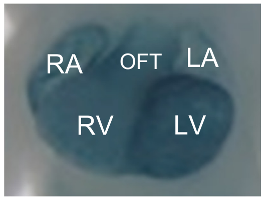	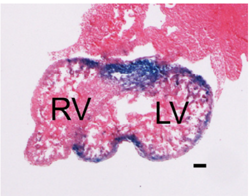	Heart (3/5)
hs2137	chr1:237,160,612-<br>237,163,517<br><br>*RYR2*	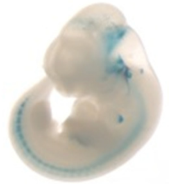	n/a	n/a	Cranial nerve (6/8),<br>Hindbrain (6/8),<br>Midbrain (7/8)
hs2138	chr1:237,174,518-<br>237,176,845<br><br>*RYR2*	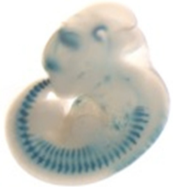	n/a	n/a	Somite (3/7)
hs2142	chr10:75,723,857-<br>75,727,727<br><br>*VCL*	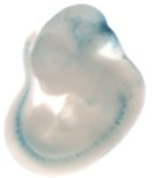	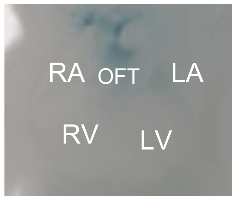	n/a	Heart (7/8),<br>Forebrain (6/8),<br>Midbrain (7/8),<br>Hindbrain (7/8),<br>Somite (8/8)
hs2143	chr10:88,443,829-<br>88,449,326<br><br>*LDB3*	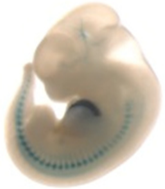	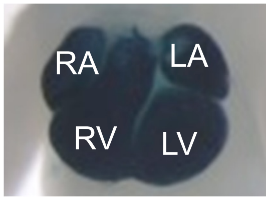	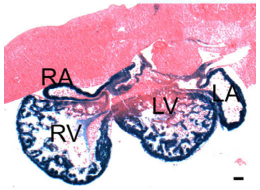	Heart (12/12),<br>Somite (9/12)

VISTA ID	Coordinates (hg19)<br>Nearest HD gene	Whole Embryo	Heart	Heart Histology	Annotation (Reproducibility)
hs2144	chr11:19,194,084-19,196,536<br>*CSRP3*	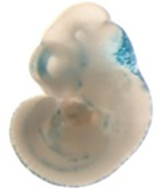	n/a	n/a	Hindbrain (3/13)
hs2145	chr11:19,227,932-19,231,643<br>*CSRP3*	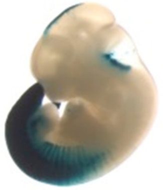	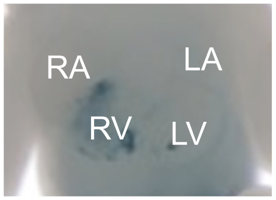	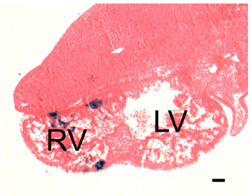	Heart (4/6),<br>Tail (5/6)
hs2151	chr12:22,044,071-22,045,600<br>*ABCC9*	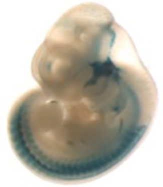	n/a	n/a	Dorsal root ganglion (5/17),<br>Trigeminal V (5/17)
hs2157	chr14:76,459,638-76,463,620<br>*TGFB3*	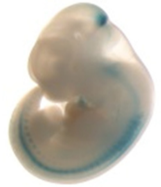	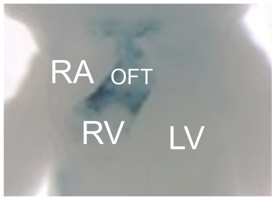	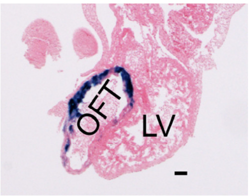	Heart (8/10)
hs2160	chr15:63,382,545-63,386,341<br>*TPM1*	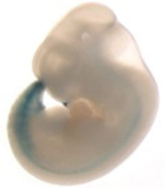	n/a	n/a	Tail (7/8)

VISTA ID	Coordinates (hg19)<br>Nearest HD gene	Whole Embryo	Heart	Heart Histology	Annotation (Reproducibility)
hs2161	chr15:73,666,606-73,670,858<br>*HCN4*	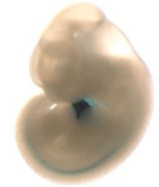	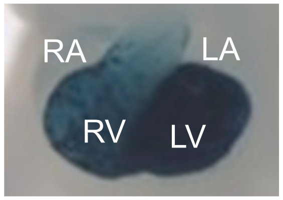	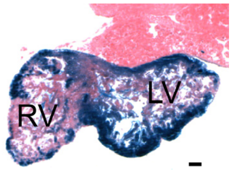	Heart (9/9)
hs2166	chr18:29,066,159-29,070,306<br>*DSG2*	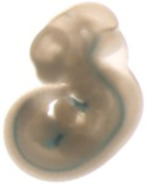	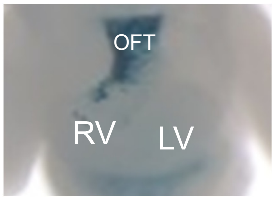	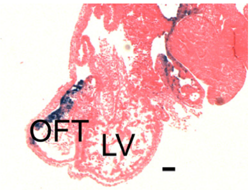	Heart (3/10), Unidentified abdominal structure (6/10)
hs2169	chr2:220,293,872-220,296,961<br>*DES*	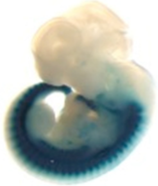	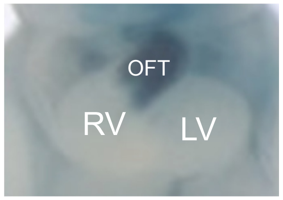	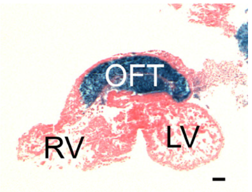	Heart (3/8)
hs2170	chr2:71,718,006-71,721,213<br>*DYSF*	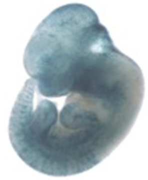	n/a	n/a	Blood vessels (3/3)
hs2173	chr20:32,009,624-32,013,185<br>*SNTA1*	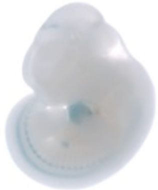	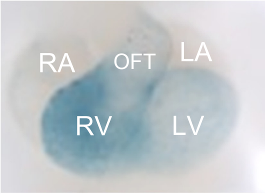	n/a	Heart (4/6)

VISTA ID	Coordinates (hg19)<br>Nearest HD gene	Whole Embryo	Heart	Heart Histology	Annotation<br>(Reproducibility)
hs2174	chr21:35,706,876-<br>35,710,787<br><br>*KCNE2*	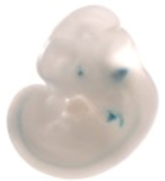	n/a	n/a	Unidentified structure next to heart (8/13)<br>Eye (11/13)<br>Hindbrain (6/13)
hs2179	chr3:52,461,982-<br>52,465,131<br><br>*TNNC1*	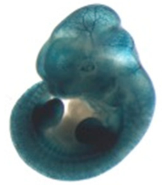	n/a	n/a	Blood vessels (5/7)
hs2181	chr5:137,029,967-<br>137,032,927<br><br>*KLHL3*	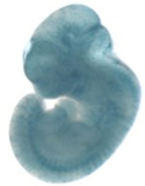	n/a	n/a	Blood vessels (9/9)
hs2185	chr5:172,692,201-<br>172,696,969<br><br>*NKX2-5*	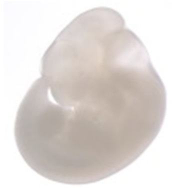	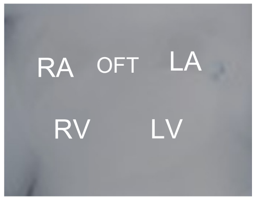	n/a	Heart (4/6)
hs2191	chr6:7,561,160-<br>7,562,735<br><br>*DSP*	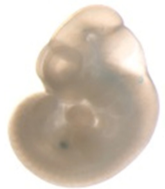	n/a	n/a	Unidentified abdominal structure, possibly pancreas (5/5)

VISTA ID	Coordinates (hg19)<br>Nearest HD gene	Whole Embryo	Heart	Heart Histology	Annotation (Reproducibility)
hs2192	chr7:150,659,986-150,663,432<br>*KCNH2*	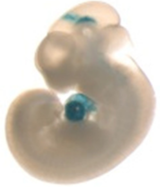	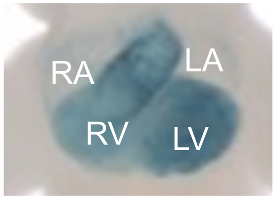	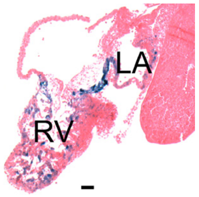	Heart (5/5)
hs2193	chr7:151,383,342-151,387,674<br>*PRKAG2*	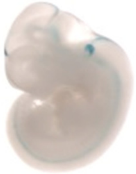	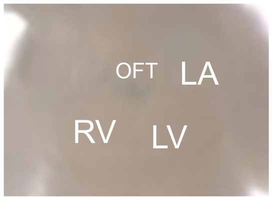	n/a	Heart (4/5)
hs2194	chr7:151,389,885-151,395,078<br>*PRKAG2*	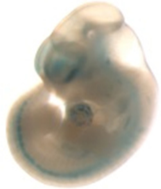	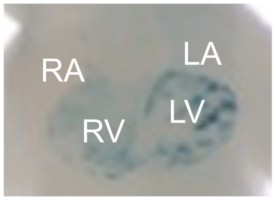	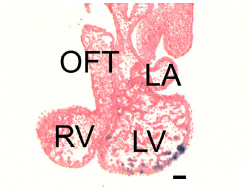	Heart (4/8),<br>Limb (3/8)
hs2199	chr7:151,450,033-151,453,713<br>*PRKAG2*	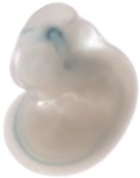	n/a	n/a	Midbrain (5/6)
hs2200	chr7:151,453,949-151,457,105<br>*PRKAG2*	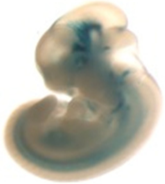	n/a	n/a	Midbrain (8/12)

VISTA ID	Coordinates (hg19)<br>Nearest HD gene	Whole Embryo	Heart	Heart Histology	Annotation<br>(Reproducibility)
hs2204	chr8:11,557,315-<br>11,561,006<br><br>*GATA4*	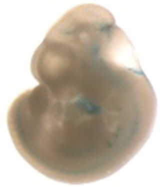	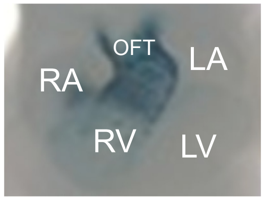	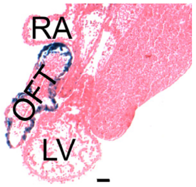	Heart (5/14)
hs2205	chr8:11,596,784-<br>11,601,556<br><br>*GATA4*	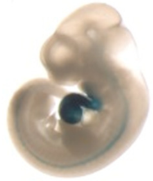	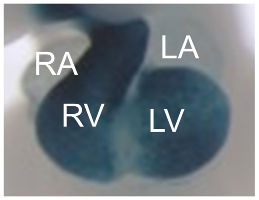	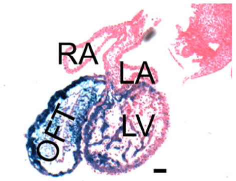	Heart (6/7)
hs2207	chr9:139,462,959-<br>139,468,347<br><br>*NOTCH1*	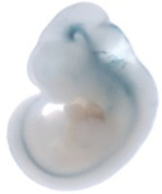	n/a	n/a	Midbrain (5/6),<br>Hindbrain (5/6),<br>Neural tube (5/6)

Representative images of whole mount E11.5 embryos for all tested enhancers active *in vivo*. For enhancers with reproducible activity in the heart, representative close-ups of the heart and heart histological sections (scale bar = 100 μm) are also shown. VISTA ID: enhancer identifier used by the VISTA Enhancer Browser (http://enhancer.lbl.gov/); HD: heart disease; Annotation: indicates tissue(s) where enhancer showed reproducible activity; Reproducibility: number of embryos with activity in given tissue over the total number of transgenic embryos; n/a: not available; RA: right atrium; LA: left atrium; RV: right ventricle; LV: left ventricle; OFT: outflow tract

Supplementary Table 7: Genotype frequency data for enhancer deletion lines

Enhancer	Litters	Total Pups Genotyped	+/+	+/-	-/-	Ratio Observed	*P*-value
mm77	45	200	50	94	56	1 : 1.9 : 1.1	0.58
mm771	33	218	48	109	61	1 : 2.3 : 1.3	0.46

Results are reported for heterozygous by heterozygous matings, which are expected to give a homozygous wild-type (+/+):heterozygous (+/-):homozygous null (-/-) ratio of 1:2:1. *P*-values were calculated using a $\chi 2$ test with 2 degrees of freedom.

Supplementary Table 8: Primers for additional transgenic assays related to enhancers mm77 and mm771

Element Name	Element Description	Genome Coordinates (Genome build name)	Primer Name	Primer Sequence*	Notes
hs1670	human *MYH7* enhancer, human homolog of mm771	chr14:23,906,587-23,908,214 (hg19)	hs1670_F	AATCAGCCCCATTGACAGAG	
			hs1670_R	CACCTCCAAACACTCCCTGAAG	
hs2265	hs1670 deletion A	chr14:23,906,587-23,908,212 minus chr14:23,907,891-23,908,116 (hg19)	hs1670_Universal_1F	**AGGGAACAAAAGCTG** AATCAGCCCCATTGACAGAG	For amplifying sequence left of deletion
			hs1670_delA_1R	TGGGAGAGTCAGCCTCCACT	
			hs1670_delA_2F	**AGGCTGACTCTCCCA** AGAGAGATGGAGGGCCGG	For amplifying sequence right of deletion
			hs1670_Universal_2R	**TGTTCCTGGAGCTCG** CCTCCAAACACTCCCTGAAG	
hs2264	hs1670 deletion B	chr14:23,906,587-23,908,212 minus chr14:23,907,369-23,907,697 (hg19)	hs1670_Universal_1F	**AGGGAACAAAAGCTG** AATCAGCCCCATTGACAGAG	For amplifying sequence left of deletion
			hs1670_delB_1R	CCCTCACTCTCCCCACAAG	
			hs1670_delB_2F	**TGGGGAGAGTGAGGG** GGAGACACCAGGGCGAATTA	For amplifying sequence right of deletion
			hs1670_Universal_2R	**TGTTCCTGGAGCTCG** CCTCCAAACACTCCCTGAAG	
hs2294	minimal region of hs1670 that retains heart activity	chr14:23,907,359-23,907,707 (hg19)	hs2294_F	**GGGGACAAGTTTGTACAAAAAAGCAGGCT** AGAGTGAGGGGCCAGGGG	
			hs2294_R	**GGGGACCACTTTGTACAAGAAAGCTGGGT** TGGTGTCTCCCTCCCTCA	
mm771	mouse homolog of hs2294	chr14:54,996,894-54,997,224 (mm10)	mm771_F	**GGGGACAAGTTTGTACAAAAAAGCAGGCT** AGAATGGGGGCCCGAACC	
			mm771_R	**GGGGACCACTTTGTACAAGAAAGCTGGGT** CAGACCCCCACCTCCCCCTA	
mm77	mouse *Myl2* enhancer	chr5:122,092,218-122,094,768 (mm10)	mm77_F	CACCGGGGGTTCCAAGGATTTAGA	
			mm77_R	GTGGCCTTACCATGACCAGT	
hs2493	human homolog of mm77	chr12:111,366,525-111,369,682 (hg19)	hs2493	CACATAAGTGCCCACCATGA	
			hs2493	GCGGCTGATCATACCGTAAT	

* **Bold** indicates homology sequence added for Gibson cloning

Supplementary Table 9: Primers for generating and validating enhancer deletions

Purpose	Primer Name	Primer Sequence	Product Size (bp)
mm771 Short arm	mm771SA.fwd	CAGTCTTGAACTAAAGGAGAACTGAGCGTG	1,508
	mm771SA.rev	GACATATACTAAAGAATTGTCTAAGTCAGCTTG	
mm771 Long arm	mm771LA.fwd	AAGGCATACTGCCTGAACCCAGTCTTAAGC	6,666
	mm771LA.rev	TCCACTAGGAATGTCACGCACGCATGCAA	
mm77 Short arm	mm77SA.fwd	CTCCAGAAGAGACTAGTGAGACAACGCA	1,346
	mm77SA.rev	TCTGATCAGGTGGGGCAAGCTTGGAAGT	
mm77 Long arm	mm77LA.fwd	GTAGCAGTTATAGTACTGAAGACCAGCTC	7,468
	mm77LA.rev	TCTAGGAAAGTCTAAATCCTTGGAACCC	
mm771 PCR screen	Bam5'-F	TTGGCTGGACGTAAACTCCTCTTCAG	2,023
	mm771.rev	CCAGATTGCCCACTTTTAAGAATATGCATAC	
mm77 PCR screen	Bam5'-F	TTGGCTGGACGTAAACTCCTCTTCAG	1,477
	mm77.rev	TACCGTTTCCCCCAGATCTGGAGAGT	
mm771 Southern probe	mm771P.fwd	GAAACAGGGCCTGAATCCAAAATGAGC	393
	mm771P.rev	GCCTATGCACAGAATGTAATTTGACTGAC	
mm77 Southern probe	mm77P.fwd	GCAGGCTCCAGTCTTGCAGATGACAA	461
	mm77P.rev	CCAAATGCCAAACCTCAGGGAAATTCAAG	
mm771 genotyping	mm771.fwd	CAAGACAAAGGGGCAGCAAGTGCTATA	wild-type: 442 bp, deletion: 193 bp

	mm771.fwd2	TGTCCAGCTGATTGTAGCAGTGGAC	
	mm771.rev2	CACGCTCAGTTCTCCTTTAGTTCAAG	
mm77 genotyping	mm77.fwd	TGTTCAAGGTAGTCCTGCGTGCCCAT	wild-type: 339 bp, deletion: 139 bp
	mm77.fwd2	GGGTTCCAAGGATTTAGACTTTCCTAGA	
	mm77.rev2	TGCGTTGTCTCACTAGTCTCTTCTGGAG	

Supplementary Table 10: Oligos for qPCR assays

Gene	IDT PrimeTime qPCR Assay	Forward Primer	Reverse Primer	Probe	Size (bp)
Myl2	Mm.PT.58.13237005	GACCATTCTCAACGCATTCAAG	GGAAAGGCTGCGAACATCT	/56-FAM/ACTGAAGGC/ZEN/TGACTATGTCCGGGA/3IABkFQ/	138
Myh7	Mm.PT.58.17465550.g	CAACATGGAGCAGATCATCAAG	CTGGTGAGGTCATTGACAGAA	/56-FAM/AAGACCAGA/ZEN/TGAATGAGCACCGGAG/3IABkFQ/	126
Nppa	Mm.PT.58.12973594.g	GGGTAGGATTGACAGGATTGG	CTCCTTGGCTGTTATCTTCGG	/56-FAM/CCAGAGTGG/ZEN/ACTAGGCTGCAACAG/3IABkFQ/	78
Nppb	Mm.PT.58.8584045.g	AGGTGACACATATCTCAAGCTG	CTTCCTACAACAACTTCAGTGC	/56-FAM/CGATCCGGT/ZEN/CTATCTTGTGCCCA/3IABkFQ/	96
Ubc	Mm.PT.58.31210189	CTGCCCTCCCACACAAAG	CTCCAGGGTGATGGTCTTAC	/56-FAM/AGATCTGCA/ZEN/TCGTCTCTCTCACGGA/3IABkFQ/	114
Actb	Mm.PT.58.33540333	GCGAGCACAGCTTCTTTG	ATGCCGGAGCCGTTGTC	/5HEX/CCGCCACCA/ZEN/GTTCGCCATG/3IABkFQ/	106

Supplementary Table 11: Antibodies for western blots

Description	Antibody	Source	Lot Number	Dilution
Myh7	NOQ7.5.4D (M8421)	Sigma-Aldrich	074M4796V	1:1,500
Myl2	EPR3741 (ab92721)	Abcam	GR121452-4	1:1,000
Gapdh	ab9484	Abcam	GR174666-4	1:1,000
Alexa-488 goat anti-mouse	A-11001	Life Technologies	1664729	1:1,000
Alexa-488 goat anti-rabbit	A-11008	Life Technologies	1583138	1:1,000

Supplementary Note 1

Previous studies showed that many heart enhancers lack the deep evolutionary conservation to non-mammalian tetrapods observed for enhancers active in other embryonic tissues, but many heart enhancers are still conserved among mammals [1,4]. For example, >50% of heart enhancers identified in mouse E11.5 heart tissue still retain sequence conservation to human [4], and >80% of candidate enhancers identified in human heart could still be aligned with the mouse genome [1]. Our results are largely consistent with these previous studies: approximately 80% of all putative enhancers identified in mouse tissue had enough sequence conservation to be mapped to the human genome (**Supplementary Table 2**). Nearly 50% of putative mouse enhancers were additionally functionally conserved in human (i.e. an H3K27ac or p300 peak is also observed in human heart tissue at the orthologous site) (**Supplementary Table 2**). This percentage of functionally conserved candidate enhancers is almost certainly an underestimate given that many enhancers active *in vivo* are specific to discrete windows during development [3], and the mouse data available are overwhelmingly from embryonic stages while the human data are almost exclusively from postnatal samples.

Supplementary Note 2

A small subset (<5%) of putative enhancers identified by the integrative analysis were >10 kb in size (**Supplementary Fig. 2a**). In particular, some of the top scoring putative enhancers are very large and overlap the bodies of genes transcribed in the heart (**Supplementary Data 2**), making such sites difficult to

interpret. However, these large putative enhancer regions should be considered for further experimental validation as they likely represent large regions containing a highly expressed gene and multiple regulatory elements, similar to collections of such sites that have been previously described [14-16]. These large putative enhancers have considerably higher average integrative analysis scores than putative enhancers <10 kb in size (**Supplementary Data 2**). Furthermore, many of these sites do contain known *in vivo* heart enhancers, as approximately half of the heart enhancers in the VISTA Enhancer Browser fall into these very large sites (**Supplementary Data 2**).

Supplementary Note 3

For all scored candidate heart enhancers, the distribution of scores is heavily weighted towards lower scores (**Fig. 2c**). This suggests that many predicted heart enhancers may be active weakly, for short time spans, or restricted to small populations of cells. However, there does appear to be a core set of predicted enhancers that have robust ChIP-seq signal in multiple heart subregions throughout development (High and Medium examples shown in **Fig. 2b**).

Scored putative heart enhancers identified by this integrative analysis overlapped 750 *in vivo* tested VISTA elements [17]. For these scored VISTA elements, those with validated heart activity (n = 152) fell into regions with scores approximately 2-fold higher than those with no heart activity (n = 598) (**Supplementary Fig. 4**) ($P < 3.8\times10^{-14}$ for all score types, by Mann-Whitney U test).

Supplementary Note 4

Dominantly inherited amino acid substitutions in *Myl2* and *Myh7* typically result in hypertrophic cardiomyopathy, and these mutations are thought to act through a dominant negative rather than loss-of-function mechanism [18]. *Myl2* loss-of-function alleles, in contrast, result in a severe, recessive form of dilated cardiomyopathy that is lethal *in utero* or in infancy [19,20]. To our knowledge, there have been no published reports of homozygous null mutations of *Myh7* in either mice or humans. Such mutations are likely embryonic lethal, but the expected form of cardiomyopathy that would result is currently unknown. To assess whether the *Δmm77* and *Δmm771* alleles result in a hypertrophic or dilated form of cardiomyopathy, we measured total heart mass and left ventricular mass to look for evidence of hypertrophy in the heart, generally, and in the left ventricle, specifically. These characteristics are distinguishing features of hypertrophic cardiomyopathy. We observed no differences in left ventricular mass or in body weight-normalized heart mass for either *Δmm77* or *Δmm771* animals (**Supplementary Fig. 14**). Therefore, the *Δmm77* and *Δmm771* mutations result in a phenotype more consistent with dilated, rather than hypertrophic, cardiomyopathy.

Supplementary References

1. May, D. *et al.* Large-scale discovery of enhancers from human heart tissue. *Nat Genet* **44,** 89–93 (2012).
2. Roadmap Epigenomics Consortium *et al.* Integrative analysis of 111 reference human epigenomes. *Nature* **518,** 317–330 (2015).
3. Nord, A. S. *et al.* Rapid and Pervasive Changes in Genome-wide Enhancer Usage during Mammalian Development. *Cell* **155,** 1521–1531 (2013).
4. Blow, M. J. *et al.* ChIP-Seq identification of weakly conserved heart enhancers. *Nat Genet* **42,** 806–810 (2010).
5. Yue, F. *et al.* A comparative encyclopedia of DNA elements in the mouse genome. *Nature* **515,** 355–364 (2014).
6. Shen, Y. *et al.* A map of the cis-regulatory sequences in the mouse genome. *Nature* **488,** 116–120 (2012).
7. van den Boogaard, M. *et al.* Genetic variation in T-box binding element functionally affects SCN5A/SCN10A enhancer. *J. Clin. Invest.* **122,** 2519–2530 (2012).
8. He, A. *et al.* Dynamic GATA4 enhancers shape the chromatin landscape central to heart development and disease. *Nat Commun* **5,** 4907 (2014).
9. Attanasio, C. *et al.* Tissue-specific SMARCA4 binding at active and repressed regulatory elements during embryogenesis. *Genome Res* **24,** 920–929 (2014).
10. Sakabe, N. J. *et al.* Dual transcriptional activator and repressor roles of TBX20 regulate adult cardiac structure and function. *Hum Mol Genet* **21,** 2194–2204 (2012).
11. Anand, P. *et al.* BET bromodomains mediate transcriptional pause release in heart failure. *Cell* **154,** 569–582 (2013).
12. van Duijvenboden, K., de Boer, B. A., Capon, N., Ruijter, J. M. & Christoffels, V. M. EMERGE: a flexible modelling framework to predict genomic regulatory elements from genomic signatures. *Nucleic Acids Res* **44,** e42 (2016).
13. Erwin, G. D. *et al.* Integrating diverse datasets improves developmental enhancer prediction. *PLoS Comput Biol* **10,** e1003677 (2014).
14. Pott, S. & Lieb, J. D. What are super-enhancers? *Nat Genet* **47,** 8–12 (2014).
15. Parker, S. C. J. *et al.* Chromatin stretch enhancer states drive cell-specific gene regulation and harbor human disease risk variants. *Proceedings of the National Academy of Sciences* **110,** 17921–17926 (2013).
16. Whyte, W. A. *et al.* Master transcription factors and mediator establish super-enhancers at key cell identity genes. *Cell* **153,** 307–319 (2013).
17. Visel, A., Minovitsky, S., Dubchak, I. & Pennacchio, L. A. VISTA Enhancer Browser--a database of tissue-specific human enhancers. *Nucleic Acids Res* **35,** D88–92 (2007).
18. Teekakirikul, P., Kelly, M. A., Rehm, H. L., Lakdawala, N. K. & Funke, B. H. Inherited cardiomyopathies: molecular genetics and clinical genetic testing in the postgenomic era. *J Mol Diagn* **15,** 158–170 (2013).

19. Weterman, M. A. J. *et al.* Recessive MYL2 mutations cause infantile type I muscle fibre disease and cardiomyopathy. *Brain* **136,** 282–293 (2013).
20. Chen, J. *et al.* Selective requirement of myosin light chain 2v in embryonic heart function. *J Biol Chem* **273,** 1252–1256 (1998).
